# Supplementary material for: Representation of genomic intratumor heterogeneity in multi-region non-small cell lung cancer patient-derived xenograft models
Source: Nat Commun. 2024 May 31;15:4653. doi: 10.1038/s41467-024-47547-3 (PMC11143323; doi:10.1038/s41467-024-47547-3)
Supplement: Supplementary file 1 — Supplementary Information [file 41467_2024_47547_MOESM1_ESM.pdf]

Supplementary Information

Representation of genomic intratumor heterogeneity in multi-region non-small cell lung cancer patient-derived xenograft models

Robert E. Hynds[*#], Ariana Huebner[*], David R. Pearce[*], Mark S. Hill, Ayse U. Akarca, David A. Moore, Sophia Ward, Kate H.C. Gowers, Takahiro Karasaki, Maise Al Bakir, Gareth A. Wilson, Oriol Pich, Carlos Martínez-Ruiz, A. S. Md Mukarram Hossain, Simon P. Pearce, Monica Sivakumar, Assma Ben Aissa, Eva Grönroos, Deepak Chandrasekharan, Krishna K. Kolluri, Rebecca Towns, Kaiwen Wang, Daniel E. Cook, Leticia Bosshard-Carter, Cristina Naceur-Lombardelli, Andrew J. Rowan, Selvaraju Veeriah, Kevin Litchfield, Philip A.J. Crosbie, Caroline Dive, Sergio A. Quezada, Sam M. Janes, Mariam Jamal-Hanjani, Teresa Marafioti, TRACERx consortium, Nicholas McGranahan[#], Charles Swanton[#]

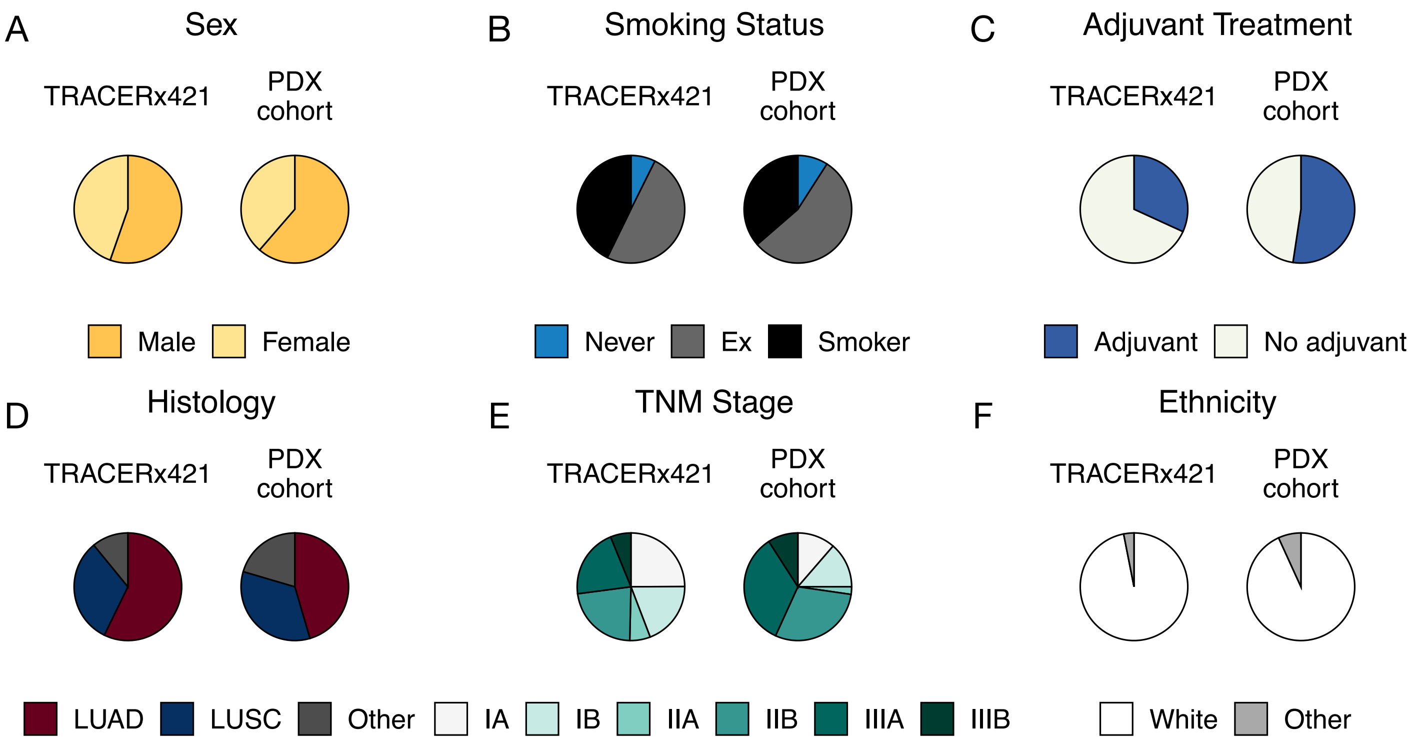

Supplementary Figure 1: PDX cohort clinical characteristics. Comparisons of the 44 patients from whom PDX models were attempted (PDX cohort) with the TRACERx421 cohort (n = 421). A) sex, B) smoking status, C) adjuvant treatment status (n = 42, as a result of two unclassifiable patients), D) histology, E) TNM stage (8th edition), and F) self-reported ethnicity.

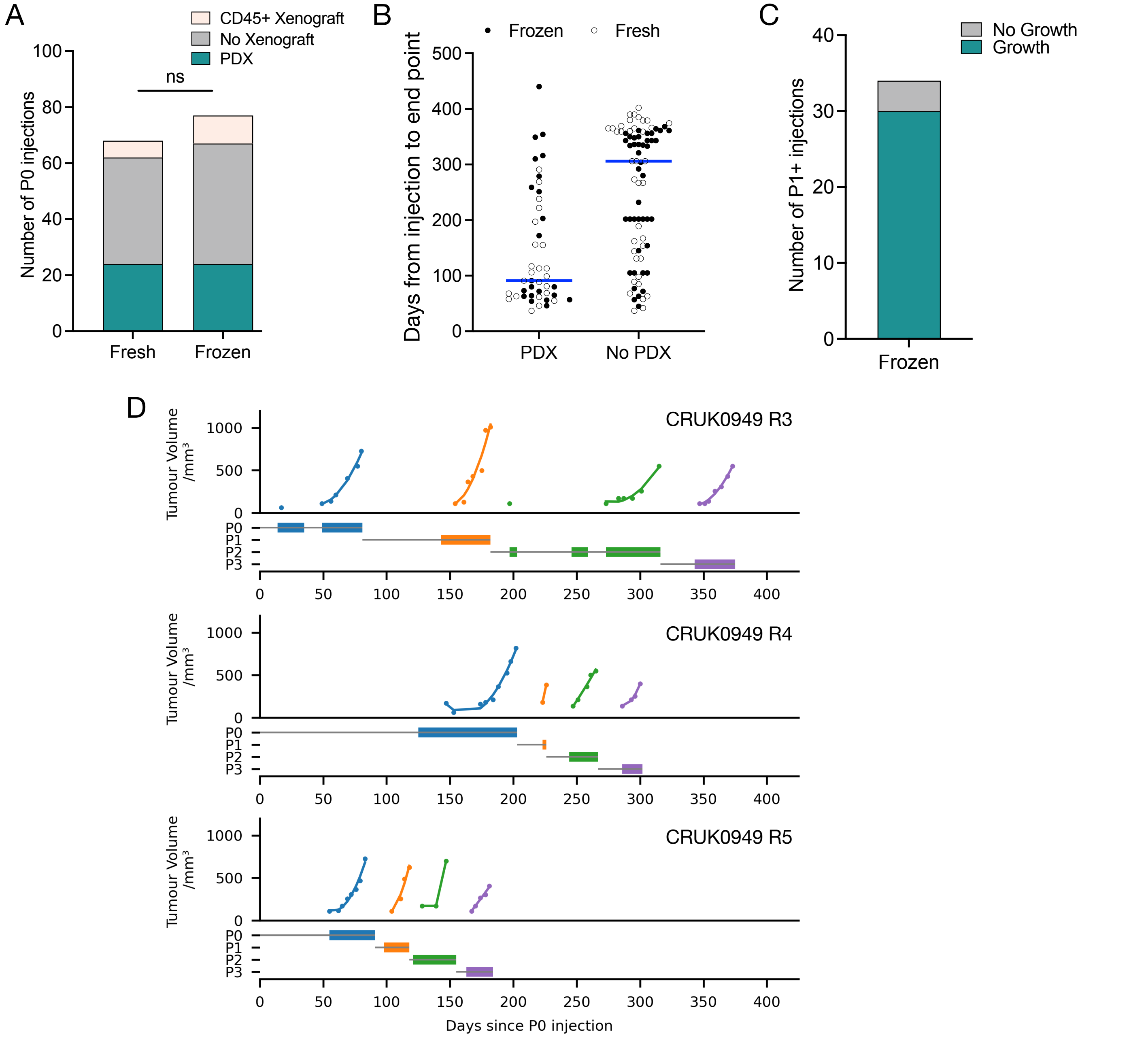

Supplementary Figure 2: Quality control and growth dynamics of PDX models. A) PDX model engraftment outcomes according to the cryopreservation status of the primary tumor material prior to injection. Chi-square test indicated no significant difference in outcomes between fresh and previously frozen injections (p = 0.686). B) Comparison of the number of days from the injection of tumor material and the harvest of a P0 xenograft between NSCLC PDX models and mice in which no xenograft was detected. C) PDX outcome (growth or no growth) from established PDX models that were cryopreserved and then re-initiated in NSG mice. D) PDX model growth curves for three region-specific PDX models from CRUK0949. Colored bars indicate timepoints where tumors were palpable. This figure panel was created using PDX-Tracker (doi.org/10.5281/zenodo.10666348; https://github.com/EpiCENTR-Lab/PDX-Tracker).

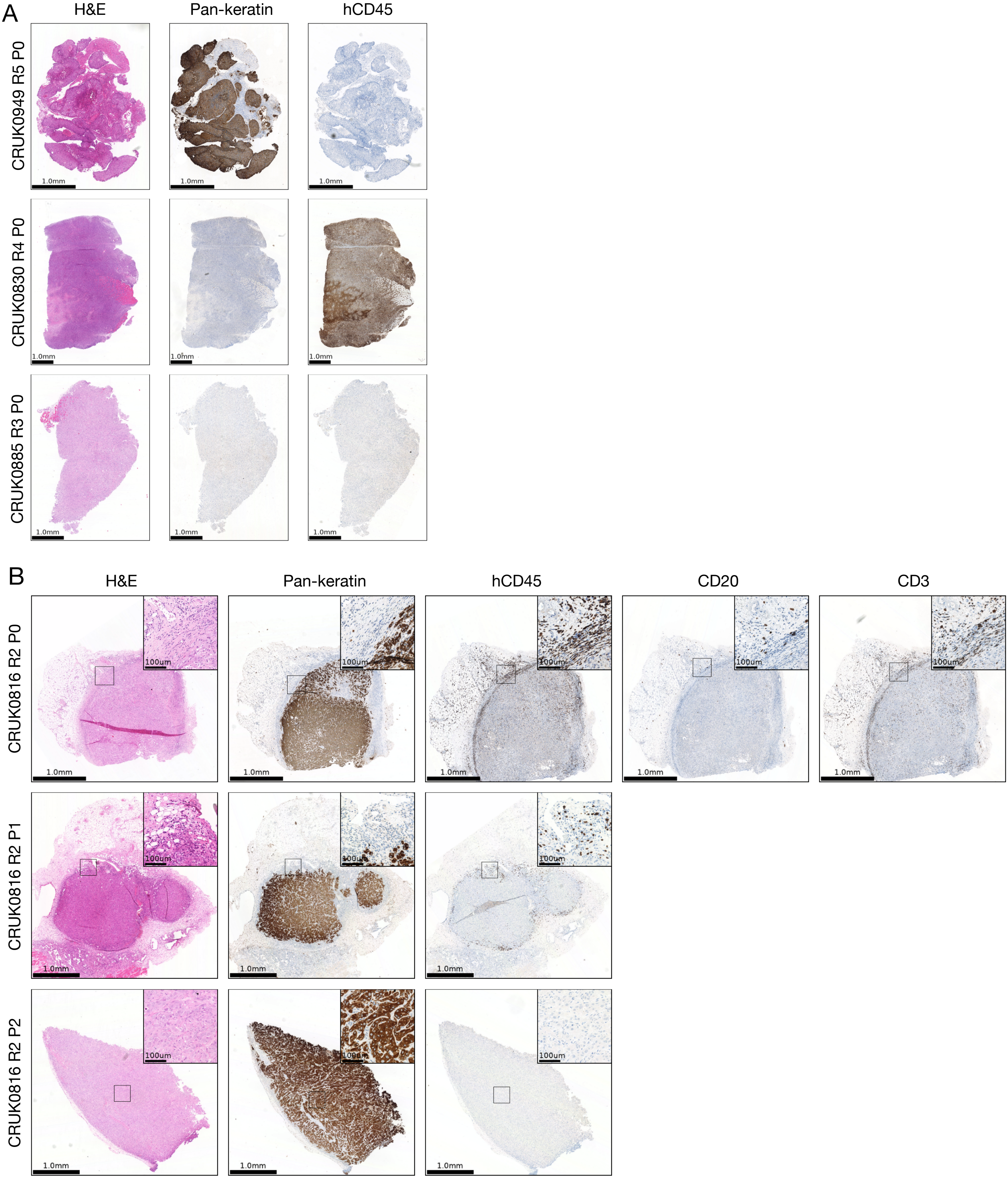

Supplementary Figure 3: Histology-based quality control in PDX models. A) Hematoxylin and eosin (H&E) staining and immunohistochemistry for pan-keratin and human CD45 (hCD45) in a representative non-small cell lung cancer xenograft (CRUK0949 R5), a representative B lymphoproliferation (CRUK0830 R4, reproduced from Pearce et al.[15]) and an exceptional case in which no reactivity to either pan-keratin or hCD45 was observed (CRUK0885 R3), due to rare tumor subtype (carcinosarcoma). Scale bars are equal to 1 mm. B) H&E staining and immunohistochemistry for pan-keratin and human CD45 (hCD45), CD20 and CD3 in the indicated passages of the CRUK0816 R2 PDX model, the only case in which hCD45+ cells were detected within the P0 NSCLC PDX tumor. Scale bars are equal to 1 mm in overview images and 100 µm in inset images. Figure panels were created and aligned using PATHOverview (doi.org/10.5281/zenodo.10678228; https://github.com/EpiCENTR-Lab/PATHOverview/).

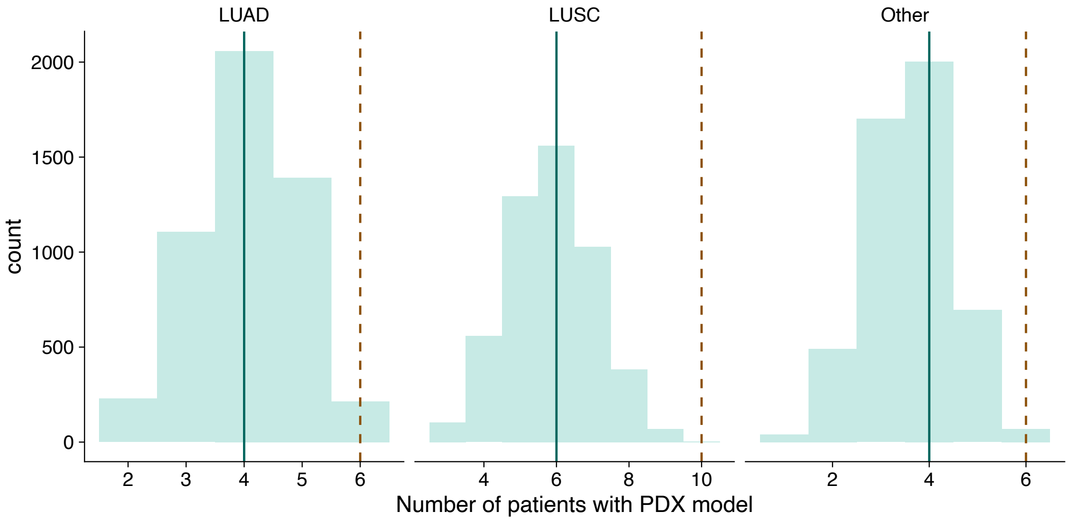

Supplementary Figure 4: Downsampling engraftment attempts split by histology. Downsampling to one engraftment attempt for each patient within three histology groups (lung adenocarcinoma, LUAD; lung squamous cell carcinoma, LUSC; other histologies). Green line indicates median modeled solution, brown dashed line indicates the observed number of patients per histology for whom PDX models were derived with a multi-region sampling approach.

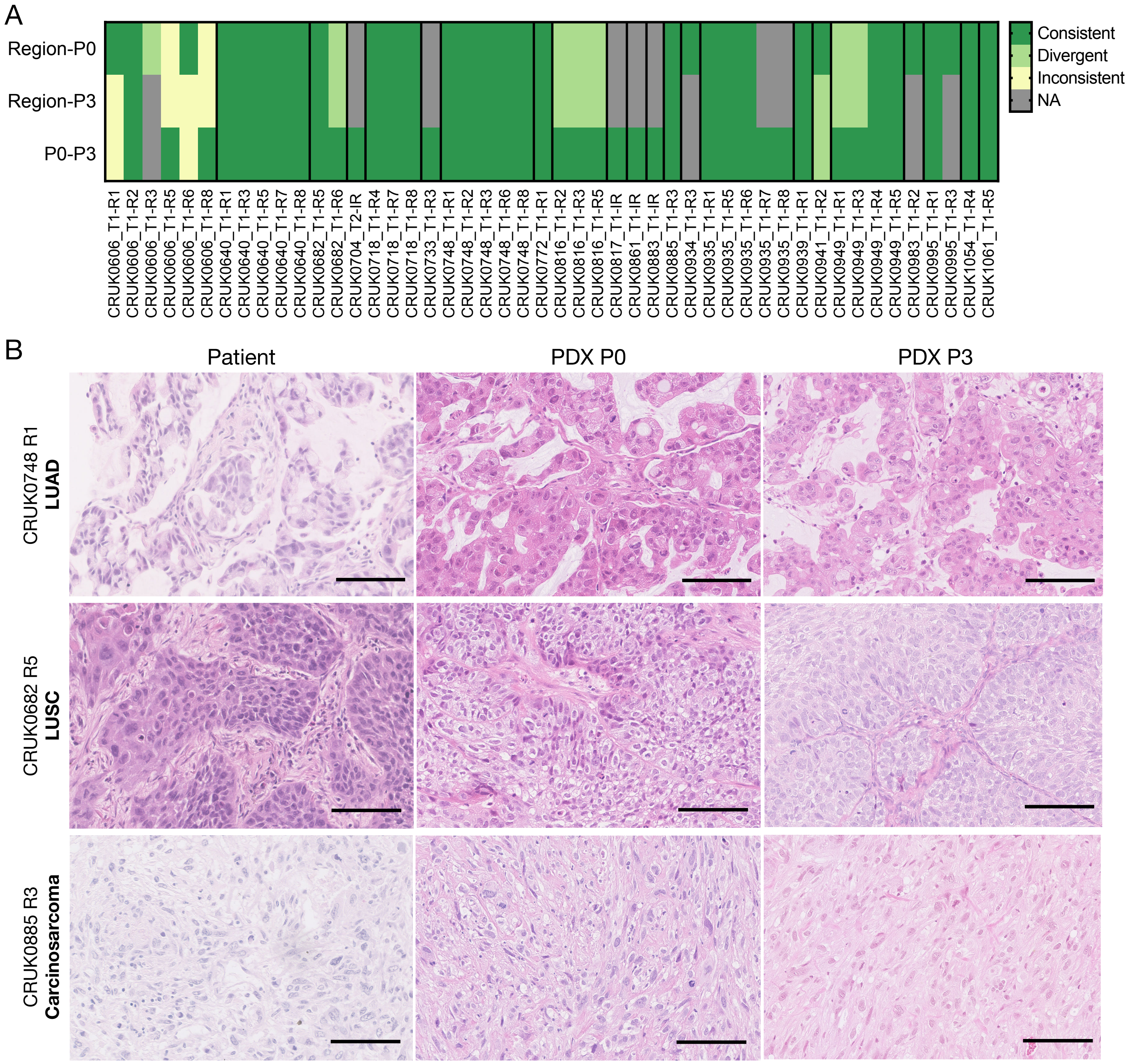

Supplementary Figure 5: Histological comparison of patient tumor regions and matched PDX models. A) Heatmap summarizing the findings of a comprehensive pathology review of patient tumor regions and matched PDX models. Tumors were classified on a three-point scale: 0/inconsistent = substantial differences that might affect the histopathological subtyping of the tumor; 1/divergent = minor differences affecting, for example, the growth pattern present within the samples; 2/consistent = samples are consistent. NA represents cases where one or more sample was unavailable for analysis (gray). B) Representative examples of hematoxylin and eosin (H&E) staining of PDX models that retained histological similarity with the patient tumor at both early (P0) and late (P3) passage. Scale bars = 100 μm.

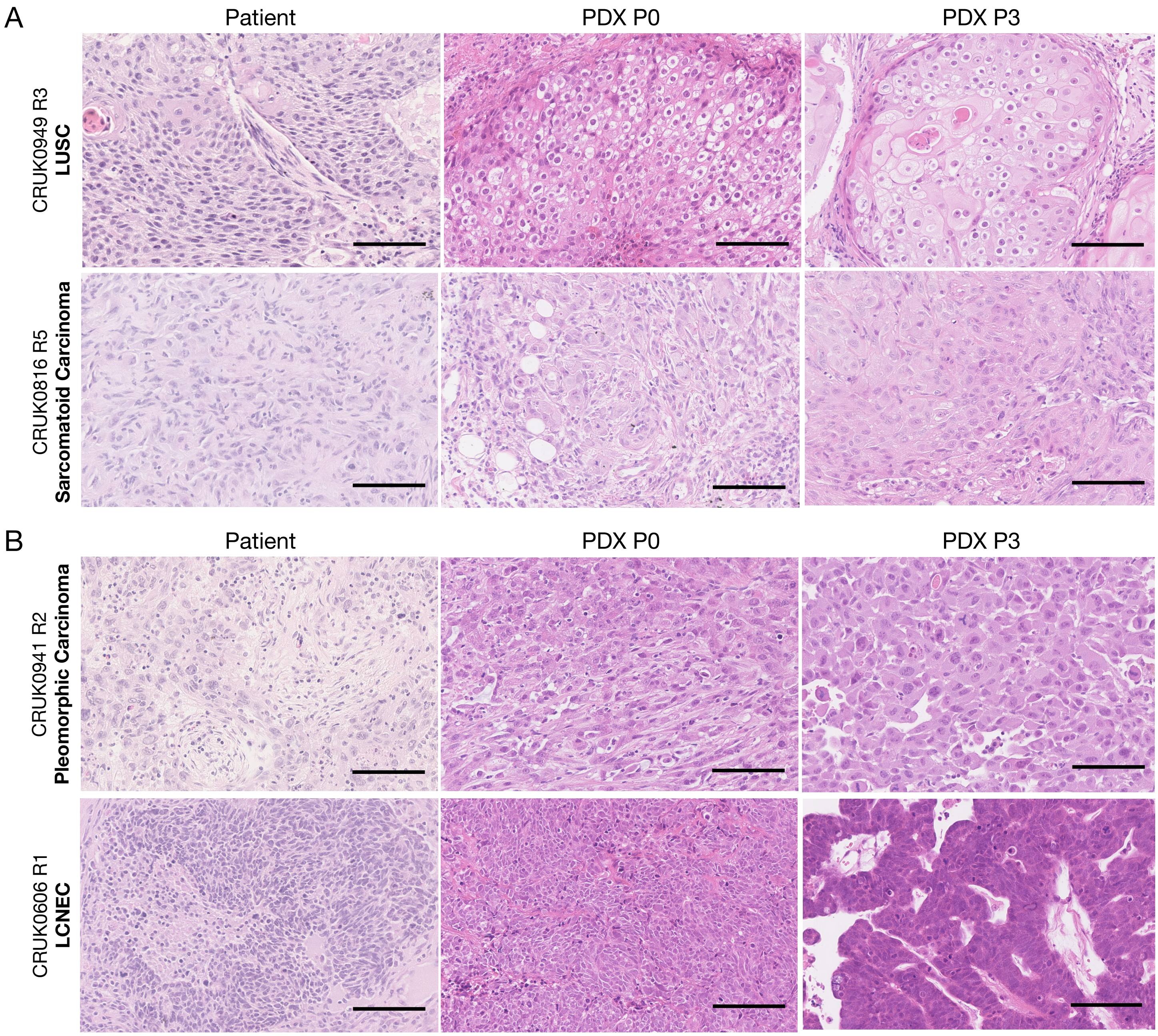

Supplementary Figure 6: Histological discrepancies between patient tumor regions and matched PDX models. A) Examples of hematoxylin and eosin (H&E) staining of PDX models whose histological appearance diverged from the matched tumor region upon model establishment (i.e. changes were evident at P0). B) Examples of H&E staining of PDX models whose histological appearance diverged from the matched tumor region in established P3 models despite having appeared similar at P0. Scale bars = 100 μm.

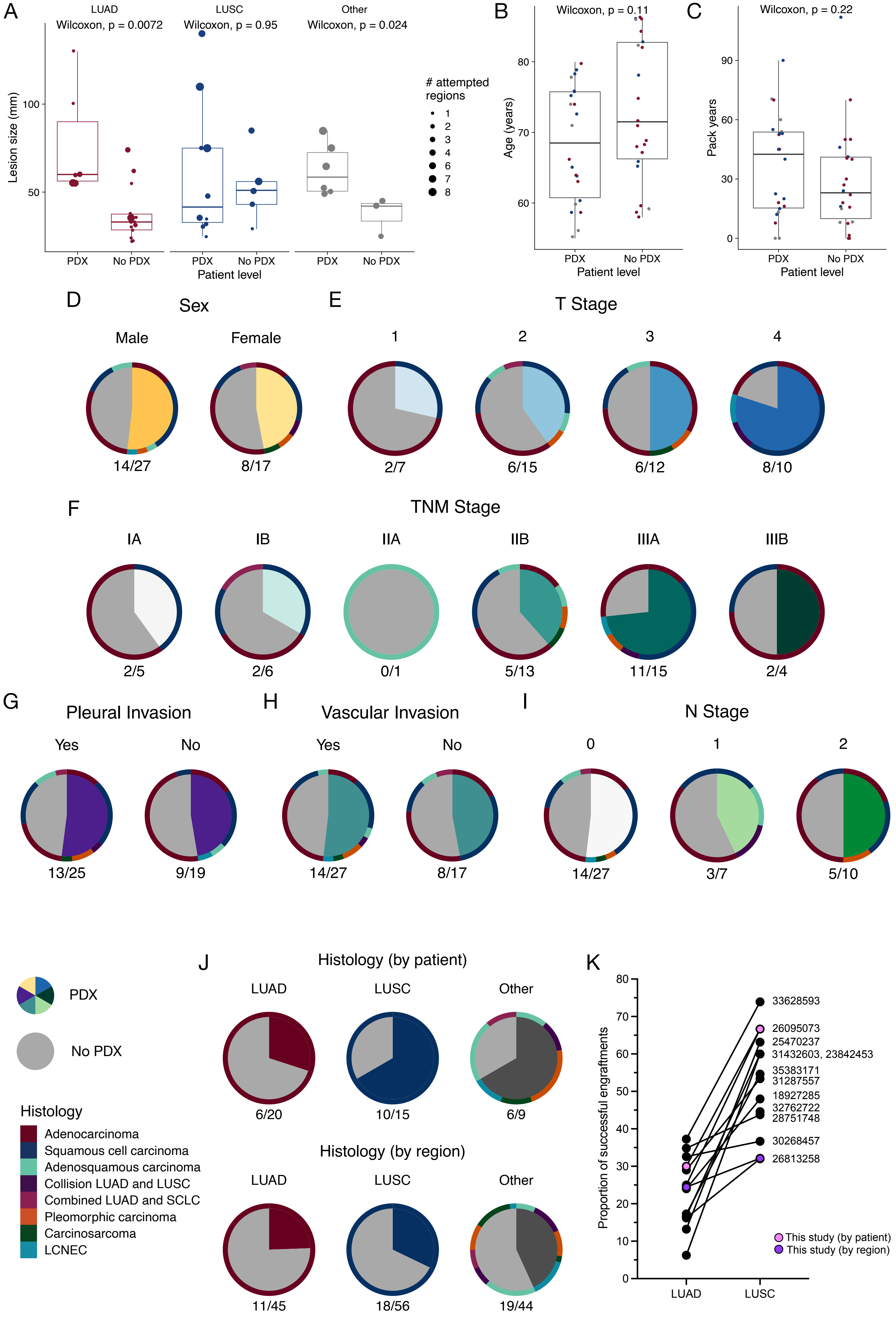
A
LUAD
Wilcoxon, p = 0.0072
LUSC
Wilcoxon, p = 0.95
Other
Wilcoxon, p = 0.024
Lesion size (mm)
attempted regions
1
2
3
4
6
7
8
PDX
No PDX
Patient level
B
Wilcoxon, p = 0.11
Age (years)
C
Wilcoxon, p = 0.22
Pack years
D
Sex
Male
Female
14/27
8/17
E
T Stage
1
2
3
4
2/7
6/15
6/12
8/10
F
TNM Stage
IA
IB
IIA
IIB
IIIA
IIIB
2/5
2/6
0/1
5/13
11/15
2/4
G
Pleural Invasion
Yes
No
13/25
9/19
H
Vascular Invasion
Yes
No
14/27
8/17
I
N Stage
0
1
2
14/27
3/7
5/10
PDX
No PDX
Histology
Adenocarcinoma
Squamous cell carcinoma
Adenosquamous carcinoma
Collision LUAD and LUSC
Combined LUAD and SCLC
Pleomorphic carcinoma
Carcinosarcoma
LCNEC
J
Histology (by patient)
LUAD
LUSC
Other
6/20
10/15
6/9
Histology (by region)
11/45
18/56
19/44
K
Proportion of successful engraftments
33628593
26095073
25470237
31432603, 23842453
35383171
31287557
18927285
32762722
28751748
30268457
26813258
This study (by patient)
This study (by region)
LUAD
LUSC

Supplementary Figure 7: Patient characteristics as predictors of PDX engraftment. A) Comparison of lesion size in mm of patient tumors that successfully generated at least one PDX model (PDX) versus those that did not generate any PDX models (no PDX) split by histological subtype of the patient tumor. Size of dots indicates the number of tumor regions for which PDX generation was attempted. Two-sided Wilcoxon rank sum test, p values as indicated. Comparison of B) patient age and C) smoking pack years with dots colored by histological subtype of the patient tumors (blue = LUSC, red = LUAD, gray = other histologies; two-sided Wilcoxon rank sum test, p values as indicated). Comparison between patient tumors that generated at least one PDX model (color) and those that did not generate any PDX models (gray) with the outer ring highlighting the histological subtype of each patient tumor for D) patient sex ($p = 1$, Fisher's exact test), E) T stage (8th edition) ($p = 0.14$, Fisher's exact test), F) TNM stage (8th edition) ($p = 0.30$, Fisher's exact test), G) pleural invasion status ($p = 1$, Fisher's exact test), H) vascular invasion status ($p = 1$, Fisher's exact test), and I) N stage ($p = 1$, Fisher's exact test). J) Comparison of histological diagnoses of patients (top) or individual regions (bottom) that generated at least one PDX model (color) versus those that did not generate any PDX models (gray) with detailed histological subtypes indicated in the outer circle (patient level engraftment success vs histology: $p = 0.064$, Fisher's exact test; region level engraftment success vs histology: $p = 0.18$, Fisher's exact test). K) Comparison of LUAD and LUSC PDX take rate by patient (pink) and region (purple) in the context of findings from prior studies (PMID). A, B, C: The box plots represent the upper and lower quartiles (box limits), the median (center line) and the whiskers span 1.5*IQR. LUAD - lung adenocarcinoma; LUSC - lung squamous cell carcinoma; SCLC - small cell lung cancer; LCNEC - large cell neuroendocrine carcinoma.

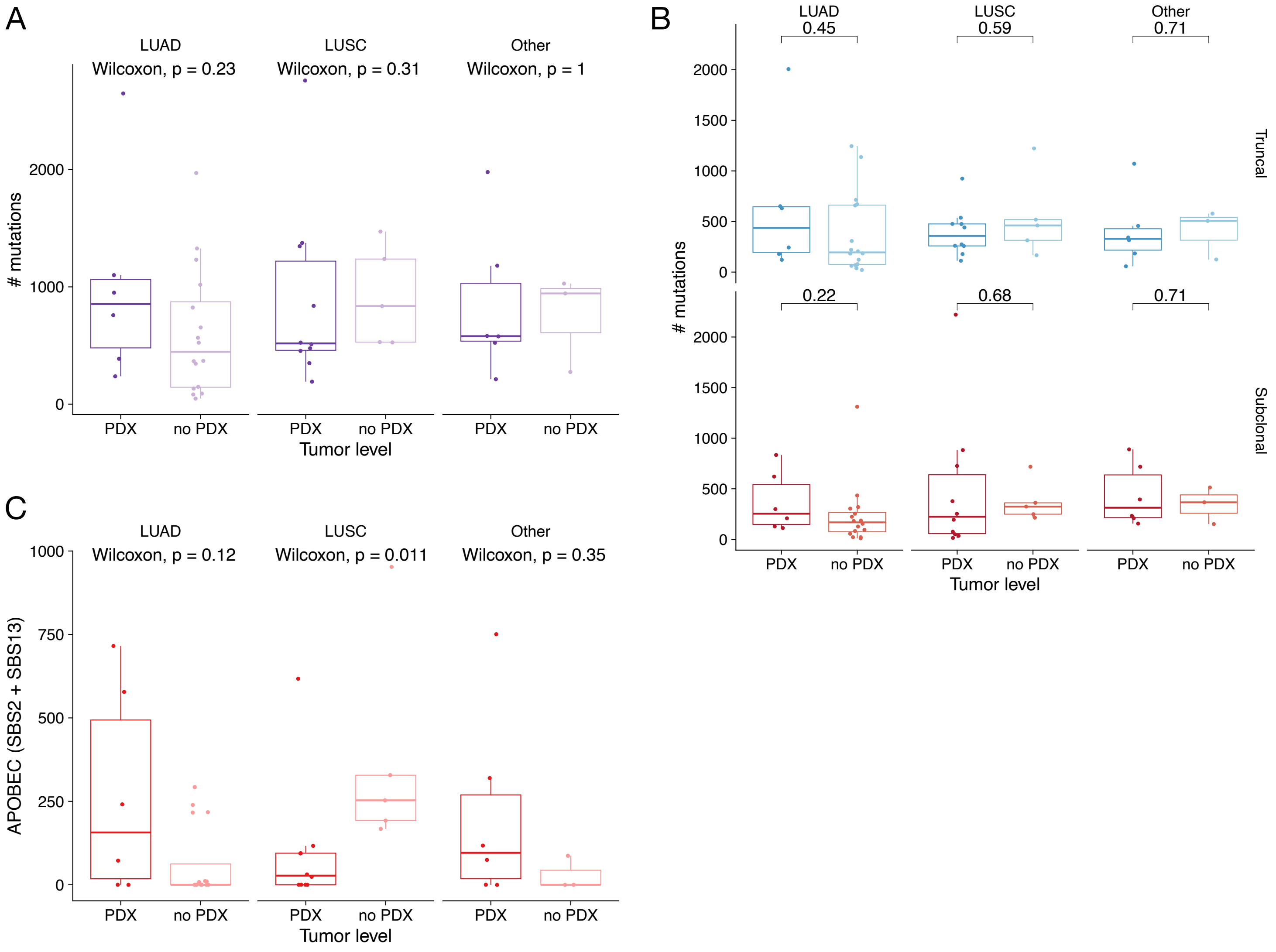

Supplementary Figure 8: Tumor mutations as predictors of PDX engraftment. A) Comparison of the total number of mutations (SNVs, multi-nucleotide variants and indels) between tumors that generated at least one PDX model (PDX) and those that did not generate any PDX model separated by tumor histological subtype. B) Comparison of the number of truncal (upper panel) and subclonal (lower panel) mutations between tumors at least one PDX model (PDX) and those that did not generate any PDX model separated by tumor histological subtype. C) Comparison of the number of mutations attributed to the APOBEC mutational signatures (SBS2 and SBS13) between tumors that generated at least one PDX model (PDX) and those that did not generate any PDX model separated by tumor histological subtype. The box plots represent the upper and lower quartiles (box limits), the median (center line) and the whiskers span 1.5*IQR. Two-sided Wilcoxon rank sum test, p values as indicated. LUAD - lung adenocarcinoma; LUSC - lung squamous cell carcinoma.

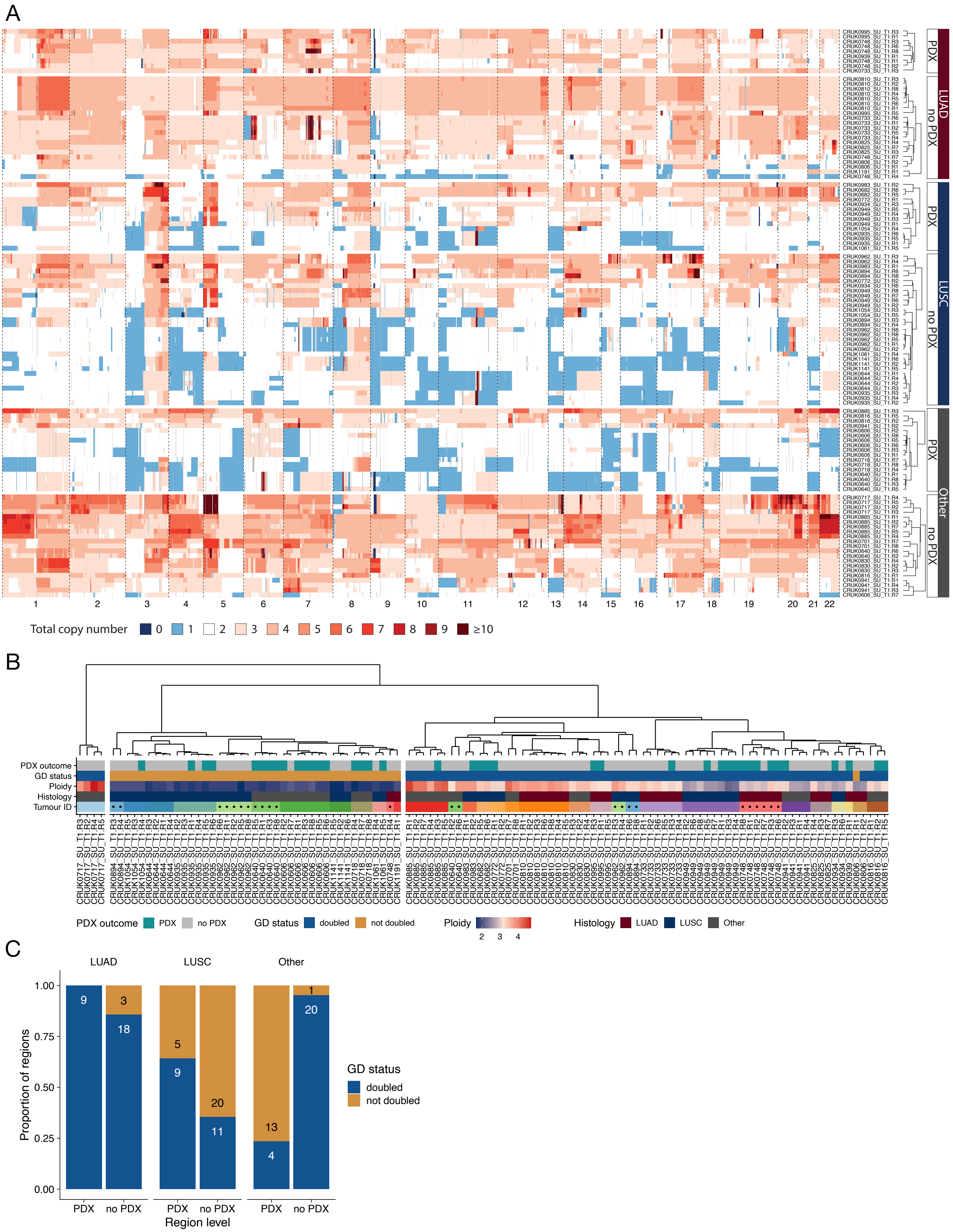

A
PDX
LUAD
no PDX
PDX
LUSC
no PDX
PDX
Other
no PDX
1 2 3 4 5 6 7 8 9 10 11 12 13 14 15 16 17 18 19 20 21 22
Total copy number 0 1 2 3 4 5 6 7 8 9 ≥10
B
PDX outcome
GD status
Ploidy
Histology
Tumour ID
PDX outcome PDX no PDX
GD status doubled not doubled
Ploidy 2 3 4
Histology LUAD LUSC Other
C
LUAD
LUSC
Other
Proportion of regions
1.00
0.75
0.50
0.25
0.00
9
3
18
5
9
20
11
13
4
1
20
PDX no PDX PDX no PDX PDX no PDX
Region level
GD status
doubled
not doubled

Supplementary Figure 9: Overview of the copy number status of primary tumor regions. A) Individual copy number segments for each tumor region are indicated along the x-axis. These have been split into minimum consistent regions across all samples, so that one column equals a single segment for all samples. The tumor regions are split by the tumor histological subtype (LUAD, LUSC, Other) as well as by PDX engraftment status (PDX, no PDX). The total copy number status (allele A + allele B) is colored. B) Dendrogram based on clustering of total copy number across the genome for each sample. The dendrogram was split into three main clusters. PDX outcome, whole-genome doubling (GD) status, ploidy and the histology are shown for each sample. The tumor of origin of each sample is highlighted in the bottom panel. Regions of the same tumor that fall into different clusters are indicated with black dots. C) Comparison of the GD status of regions that generated a PDX model and those that did not. The number of regions in each group are shown in the plot. P = 0.23, overall Fisher's exact test; LUAD, p = 0.53; LUSC, p = 0.11; Other, p = 5.24e-6. LUAD - lung adenocarcinoma; LUSC - lung squamous cell carcinoma.

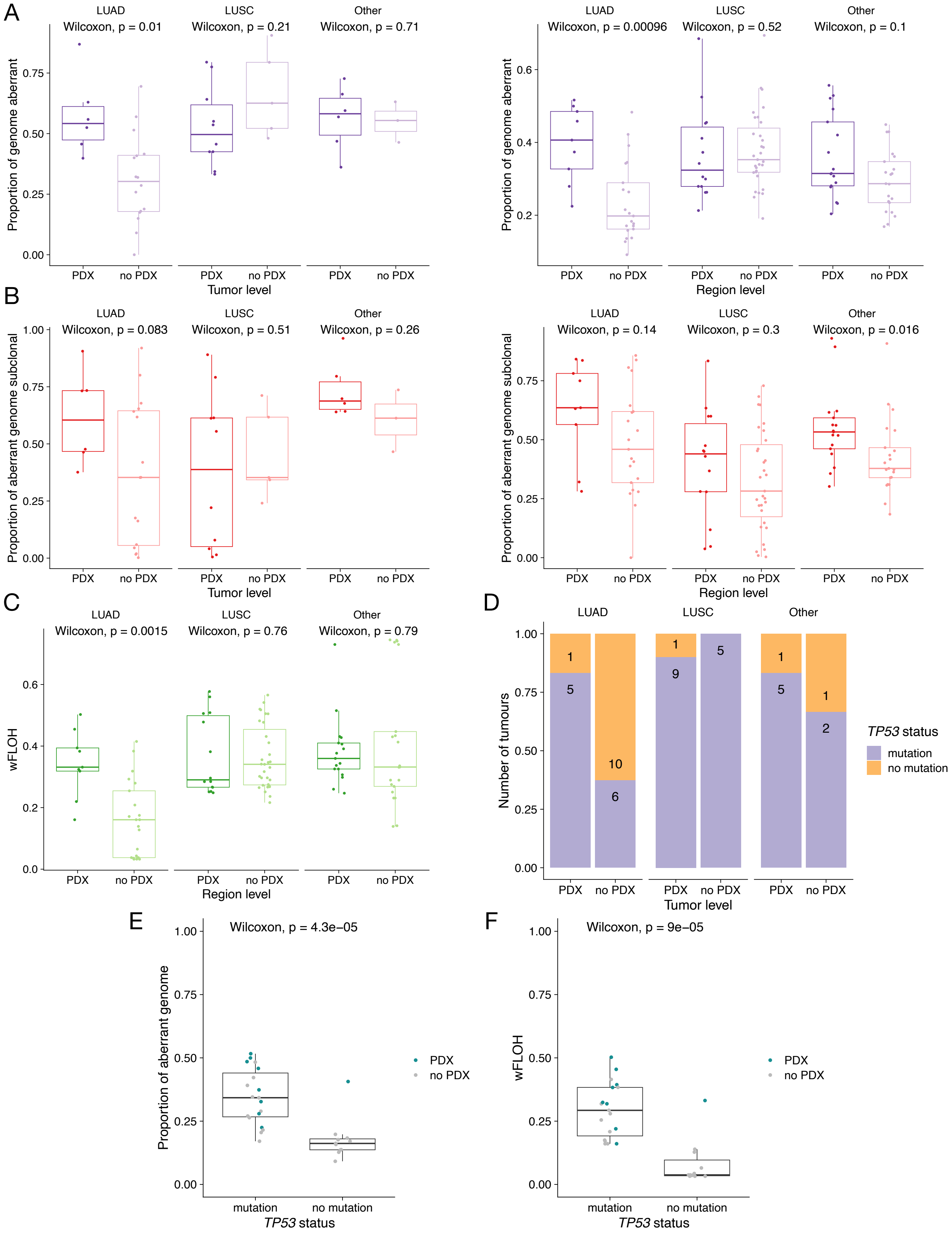
A
LUAD
Wilcoxon, p = 0.01
LUSC
Wilcoxon, p = 0.21
Other
Wilcoxon, p = 0.71
Proportion of genome aberrant
0.00
0.25
0.50
0.75
PDX
no PDX
Tumor level
LUAD
Wilcoxon, p = 0.00096
LUSC
Wilcoxon, p = 0.52
Other
Wilcoxon, p = 0.1
0.2
0.4
0.6
Region level
B
LUAD
Wilcoxon, p = 0.083
LUSC
Wilcoxon, p = 0.51
Other
Wilcoxon, p = 0.26
Proportion of aberrant genome subclonal
1.00
Tumor level
LUAD
Wilcoxon, p = 0.14
LUSC
Wilcoxon, p = 0.3
Other
Wilcoxon, p = 0.016
Region level
C
LUAD
Wilcoxon, p = 0.0015
LUSC
Wilcoxon, p = 0.76
Other
Wilcoxon, p = 0.79
wFLOH
0.0
Region level
D
Number of tumours
1
5
10
6
9
2
TP53 status
mutation
no mutation
Tumor level
E
Wilcoxon, p = 4.3e−05
Proportion of aberrant genome
mutation
no mutation
TP53 status
F
Wilcoxon, p = 9e−05
wFLOH

Supplementary Figure 10: Tumor copy number alterations as predictors of PDX engraftment. Comparison of PDX success for A) proportion of the genome that is aberrant, B) proportion of the aberrant genome that is subclonal, split by histology subtype at the tumor level (left) or region level (right). C) Comparison of the weighted fraction of the genome subject to loss of heterozygosity (wFLOH) between tumors that generated at least one PDX model and those that did not generate any PDX model at the region level. D) Comparison of the *TP53* status of tumors that generated at least one PDX model (PDX) and those that did not generate any PDX model separated by tumor histological subtype ($p = 0.026$, overall Fisher's exact test; LUAD, $p = 0.15$; LUSC, $p = 1$; Other, $p = 1$). The number of tumors in each group are shown in the plot. E) Proportion of the genome that is aberrant for LUAD tumors split by *TP53* mutation status. Colors indicate whether the region successfully engrafted (PDX - teal) or did not give rise to a PDX model (no PDX - gray). F) wFLOH of LUAD tumors split by *TP53* mutation status. Colors indicate whether the region successfully engrafted (PDX, teal) or did not give rise to a PDX model (no PDX, gray). The box plots represent the upper and lower quartiles (box limits), the median (center line) and the whiskers span 1.5*IQR. Two-sided Wilcoxon rank sum test, p values as indicated. LUAD - lung adenocarcinoma; LUSC - lung squamous cell carcinoma.

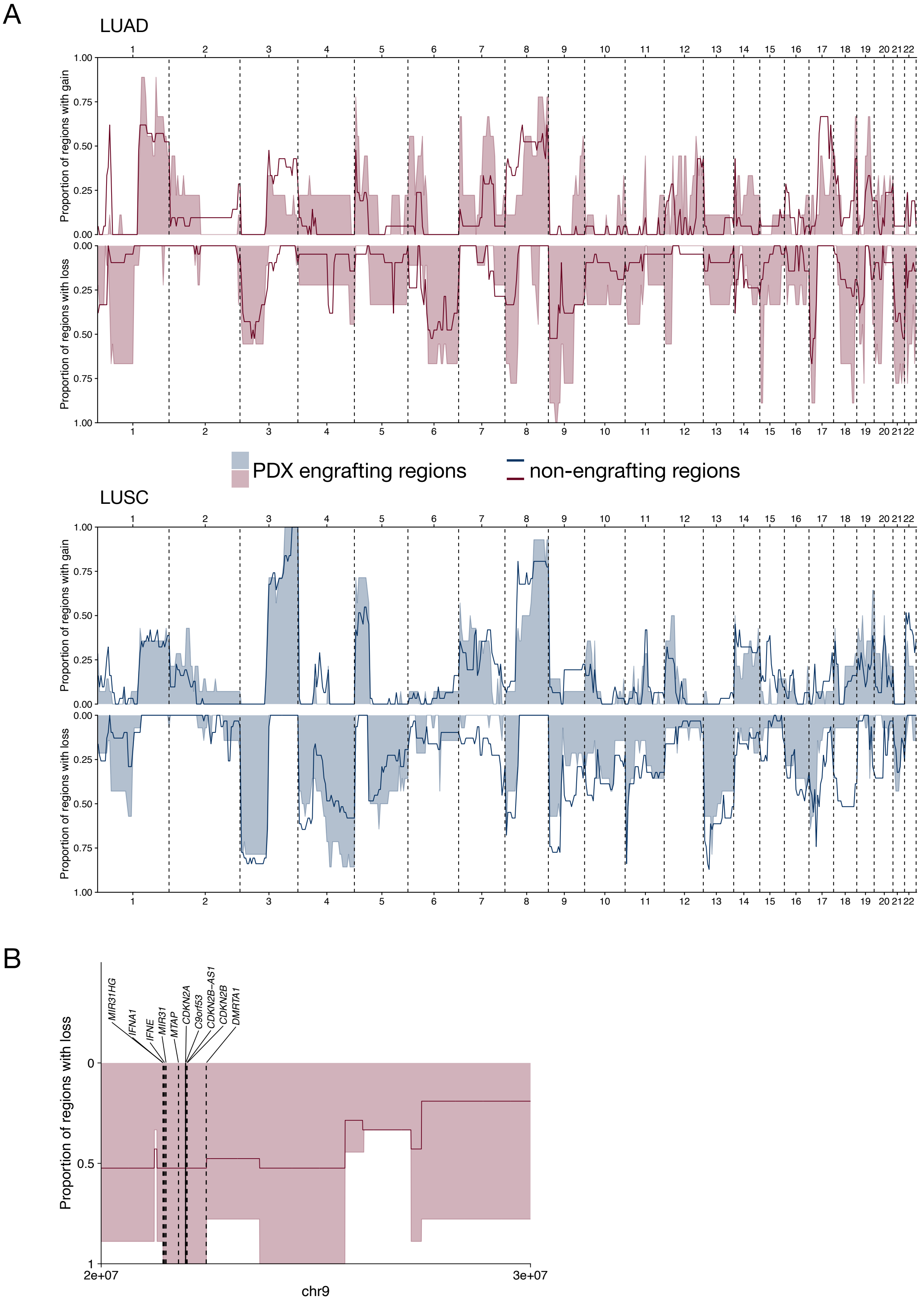

Supplementary Figure 11: Investigation of gain/loss events in tumor regions that gave rise to PDX models. A) Cross-genome plot highlighting proportion of tumor regions with a gain or loss of a particular genomic region in LUAD (red) and LUSC (blue) tumors. The x-axis is separated into 5 Mb bins and a sample is considered to have a gain (or loss) in a bin if any segment overlapping the bin is gained (or lost). The dark line represents data from non-engrafting primary tumor samples (n LUAD = 21, n LUSC = 31), while the shaded area represents data from regions that engrafted (n LUAD = 9, n LUSC = 14). B) Higher resolution of the proportion of LUAD samples with a loss on chromosome 9 between positions 20 and 30 million. The x-axis is separated into 5 kb bins and a sample is considered to have a gain (or loss) in a bin if any segment overlapping the bin is gained (or lost). The highlighted genes were found within the same segment in all LUAD tumor regions that engrafted (n = 9). LUAD - lung adenocarcinoma; LUSC - lung squamous cell carcinoma.

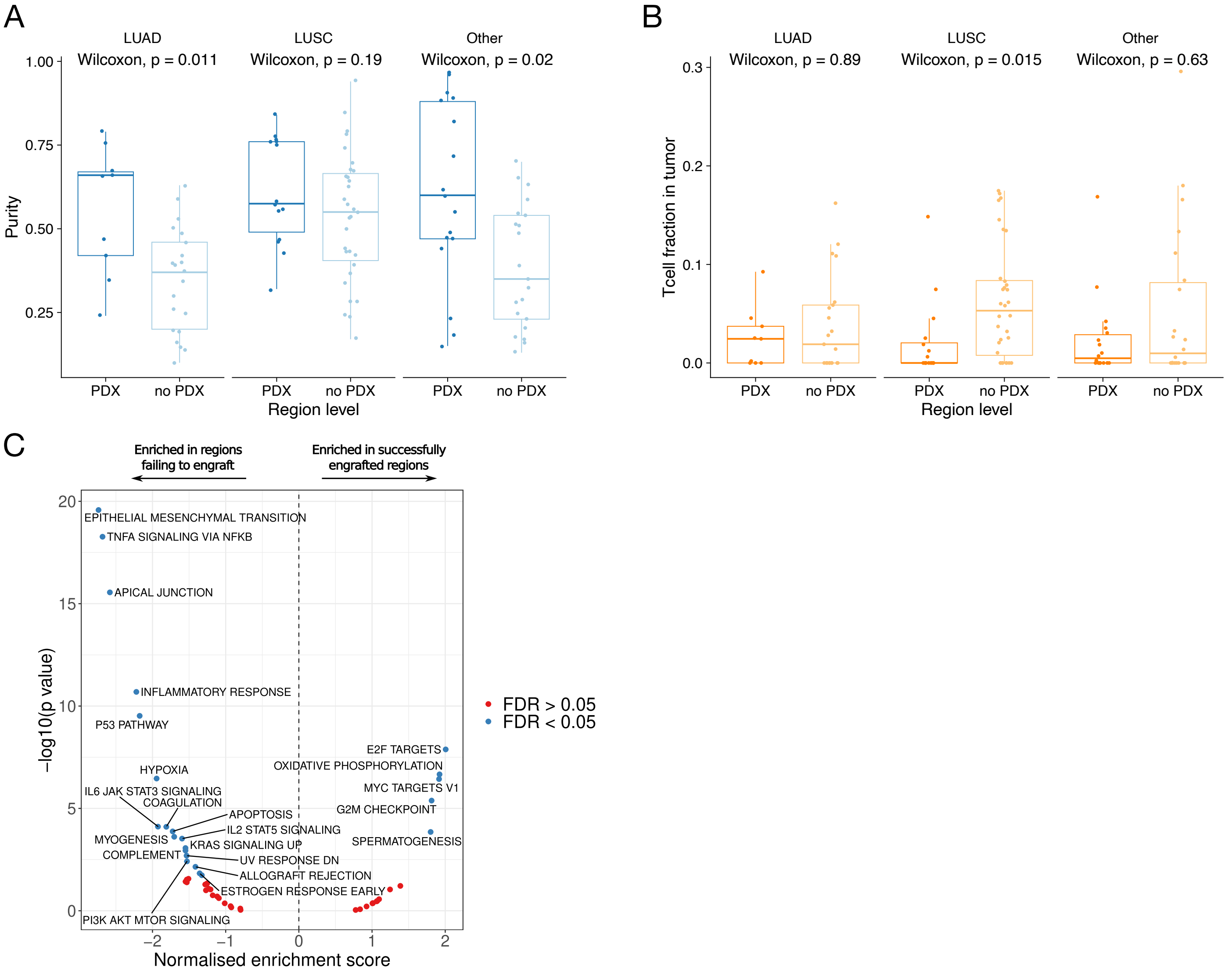

Supplementary Figure 12: Other predictors of PDX engraftment. A) Comparison of the tumor purity between regions that generated a PDX model (PDX) and those that did not generate a PDX model (no PDX) separated by tumor histological subtype. Sample purity was derived from whole-exome sequencing data using ASCAT and represents the proportion of tumor cells as a total of all cells after the removal of mouse reads. B) Comparison of the adjusted T cell fraction between regions that generated a PDX model and those that did not generate a PDX model separated by tumor histological subtype. T cell fraction was estimated using the T Cell Exome TREC Tool (T Cell ExTRECT). A,B: The box plots represent the upper and lower quartiles (box limits), the median (center line) and the whiskers span 1.5*IQR. Two-sided Wilcoxon rank sum test, p values as indicated. C) Gene set enrichment analysis (GSEA) of functional groups from hallmark gene sets between regions that generated a PDX model and those that did not. Only tumors with RNA-seq data available in the TRACERx 421 dataset were included (32 primary tumor samples, 10 PDX, 22 no PDX samples from 8 patients). Dots are colored by significance of enrichment after FDR correction, with blue dots indicating significant ($p < 0.05$) and red dots indicating not significant ($p > 0.05$). Mean normalized enrichment score (NES) is displayed on the x-axis and indicates the enrichment for a given gene set, and the negative log of the adjusted p-value is displayed on the y-axis. A positive NES indicates enrichment of the pathway in successfully engrafted regions, whereas a negative value indicates enrichment in regions that failed to engraft as PDX models. LUAD - lung adenocarcinoma; LUSC - lung squamous cell carcinoma.

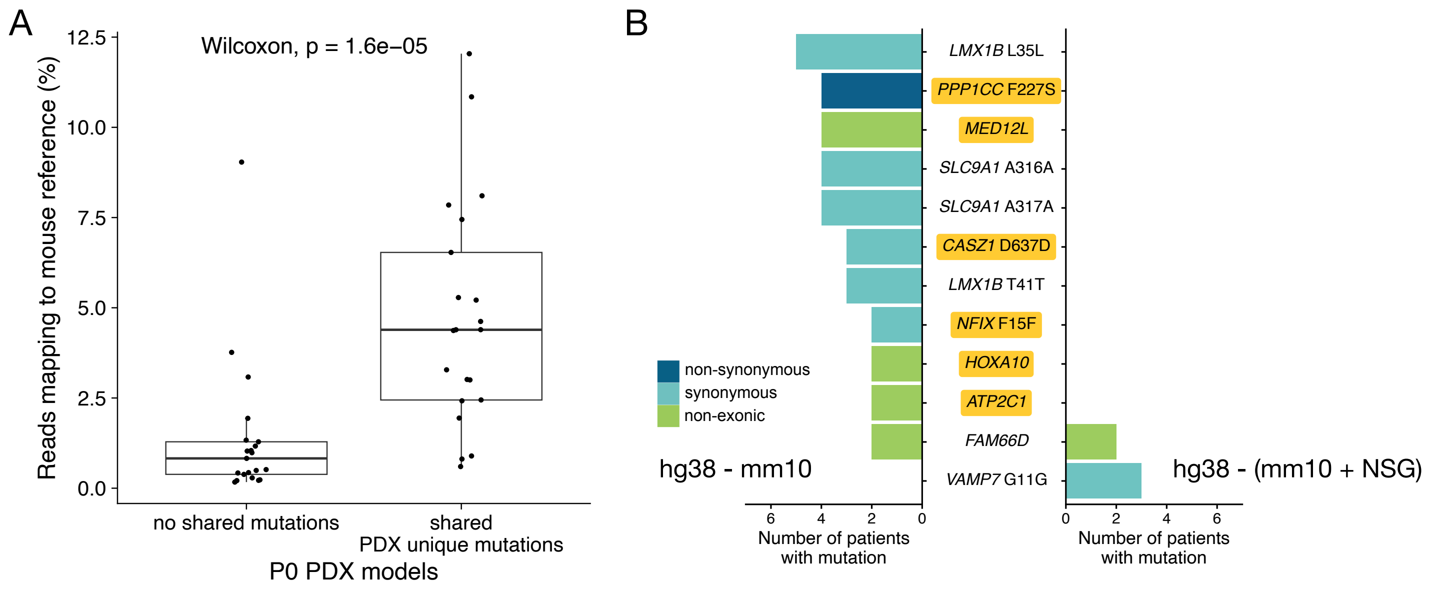

Supplementary Figure 13: Investigation and validation of an NSG-adapted reference genome. A) The proportion of reads mapping to the mm10 reference genome as calculated by FastQ Screen in whole-exome sequenced (WES) samples from P0 PDX models. WES samples with shared mutations are those containing at least one of the remaining non-zero mutations as shown on the right-hand side of Figure 3B (i.e. *PPP2R5A* S36I and below). Samples with no shared mutations are either those that are unaffected by this issue entirely, or where all shared mutations were successfully removed by using the NSG-adapted reference genome. The box plot represents the upper and lower quartiles (box limits), the median (center line) and the whiskers span 1.5*IQR. Two-sided Wilcoxon rank sum test, p value as indicated. B) Overview of shared mutations found in two or more NSCLC xenografts derived from an independent cohort of seven non-small cell lung cancer (NSCLC) patients using the mm10 reference genome (GRCm38; left) or the NSG-adapted reference genome (right). Mutations that were also found in the initial analysis shown in Figure 3B are highlighted in yellow.

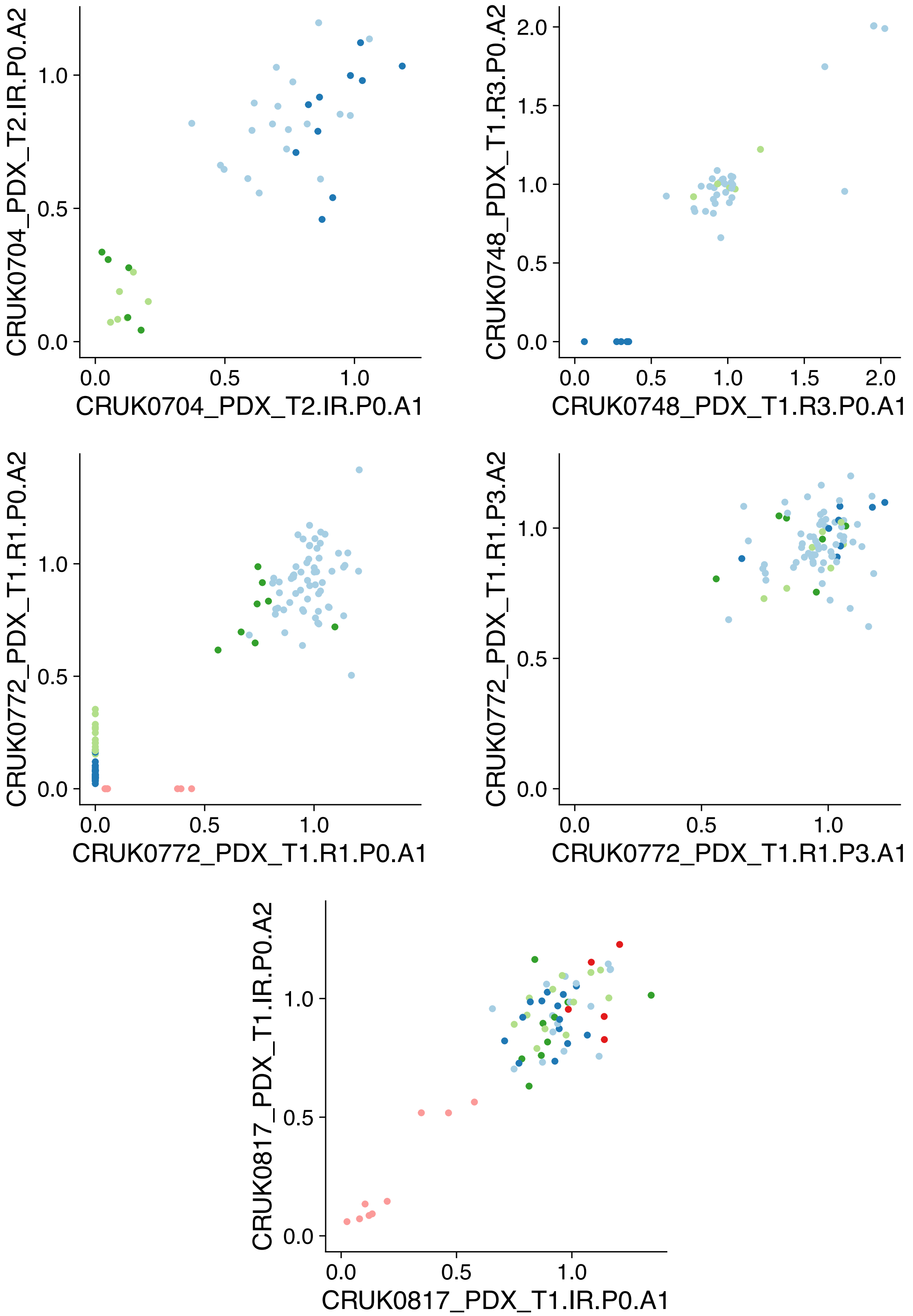

Supplementary Figure 14: Intra-PDX model genomic heterogeneity. Plots show the cancer cell fraction of spatially distinct samples from the same PDX model tumor (i.e. represent a comparison of intra-PDX model heterogeneity). Dots represent individual mutations and are colored by mutation cluster. Mutations from subclonal clusters present in either sample are shown.

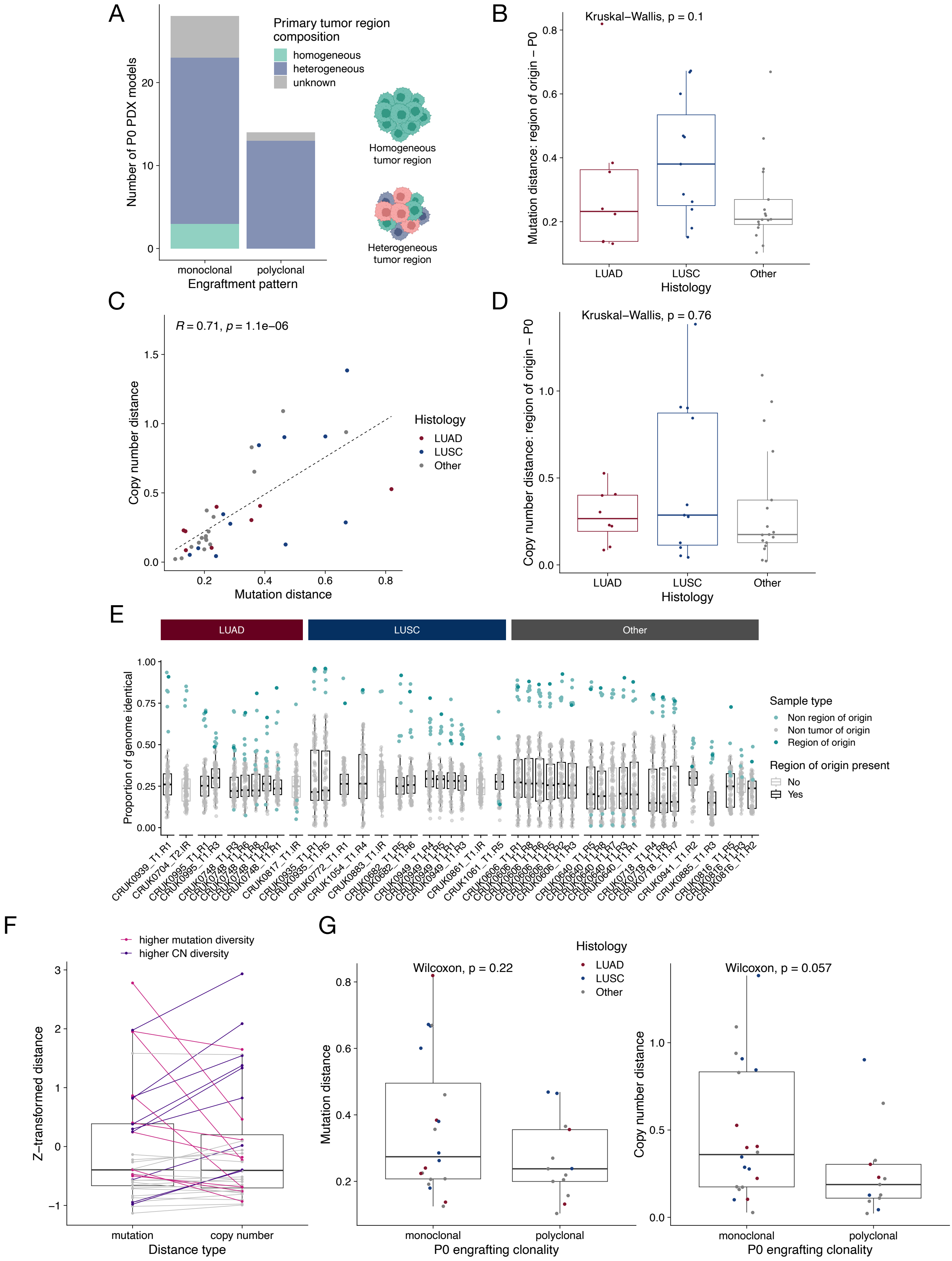
A
Number of P0 PDX models
Primary tumor region composition
homogeneous
heterogeneous
unknown
Homogeneous tumor region
Heterogeneous tumor region
monoclonal
polyclonal
Engraftment pattern
B
Kruskal–Wallis, p = 0.1
Mutation distance: region of origin – P0
LUAD
LUSC
Other
Histology
C
R = 0.71, p = 1.1e–06
Copy number distance
Mutation distance
Histology
LUAD
LUSC
Other
D
Kruskal–Wallis, p = 0.76
Copy number distance: region of origin – P0
LUAD
LUSC
Other
Histology
E
LUAD
LUSC
Other
Proportion of genome identical
Sample type
Non region of origin
Non tumor of origin
Region of origin
Region of origin present
No
Yes
F
higher mutation diversity
higher CN diversity
Z–transformed distance
mutation
copy number
Distance type
G
Histology
LUAD
LUSC
Other
Wilcoxon, p = 0.22
Mutation distance
monoclonal
polyclonal
P0 engrafting clonality
Wilcoxon, p = 0.057
Copy number distance
monoclonal
polyclonal
P0 engrafting clonality

Supplementary Figure 15: Genomic characteristics of early passage PDX models. A) Comparison of clonal composition of primary tumor regions giving rise to PDX models that are monoclonal or polyclonal with respect to their primary tumor region. B) Mutational distance of the region of origin to P0 PDX models split by tumor histological subtypes. Two-sided Kruskal-Wallis test, p value as indicated. C) Correlation between mutational distance and copy number distance of region of origin to P0 PDX models. Each point represents one PDX model and points are colored by tumor histology. The black dashed line represents a linear model fitted to the points. D) Copy number distance of the regions of origin to P0 PDX models split by tumor histological subtypes. Two-sided Kruskal-Wallis test, p value as indicated. E) Proportion of genome identical between the P0 PDX model (indicated on the x-axis) and the region of origin (teal), other regions of the same primary tumor (light teal), and tumor regions of other primary tumors within the cohort (gray). P0 PDX samples are grouped based on the tumor of origin and ordered by the tumor histology. The boxplot shows the distribution of the proportion genome identical between the P0 PDX model and tumor regions from other primary tumors that generated at least one PDX model (n tumors = 19, n regions = 103). PDX models where the box plots are highlighted in gray do not have a matched region of origin. F) Comparison of z-transformed mutation and copy number distance. Pink and purple lines highlight the highest and lowest quartiles, respectively, of the difference between mutational and copy number diversity. G) Comparison of mutational distance (left) and copy number distance (right) between region of origin and P0 for monoclonal versus polyclonal P0 PDX models. Two-sided Wilcoxon rank sum test, p values as indicated. The box plots represent the upper and lower quartiles (box limits), the median (center line) and the whiskers span 1.5*IQR. LUAD - lung adenocarcinoma; LUSC - lung squamous cell carcinoma; CN - copy number.

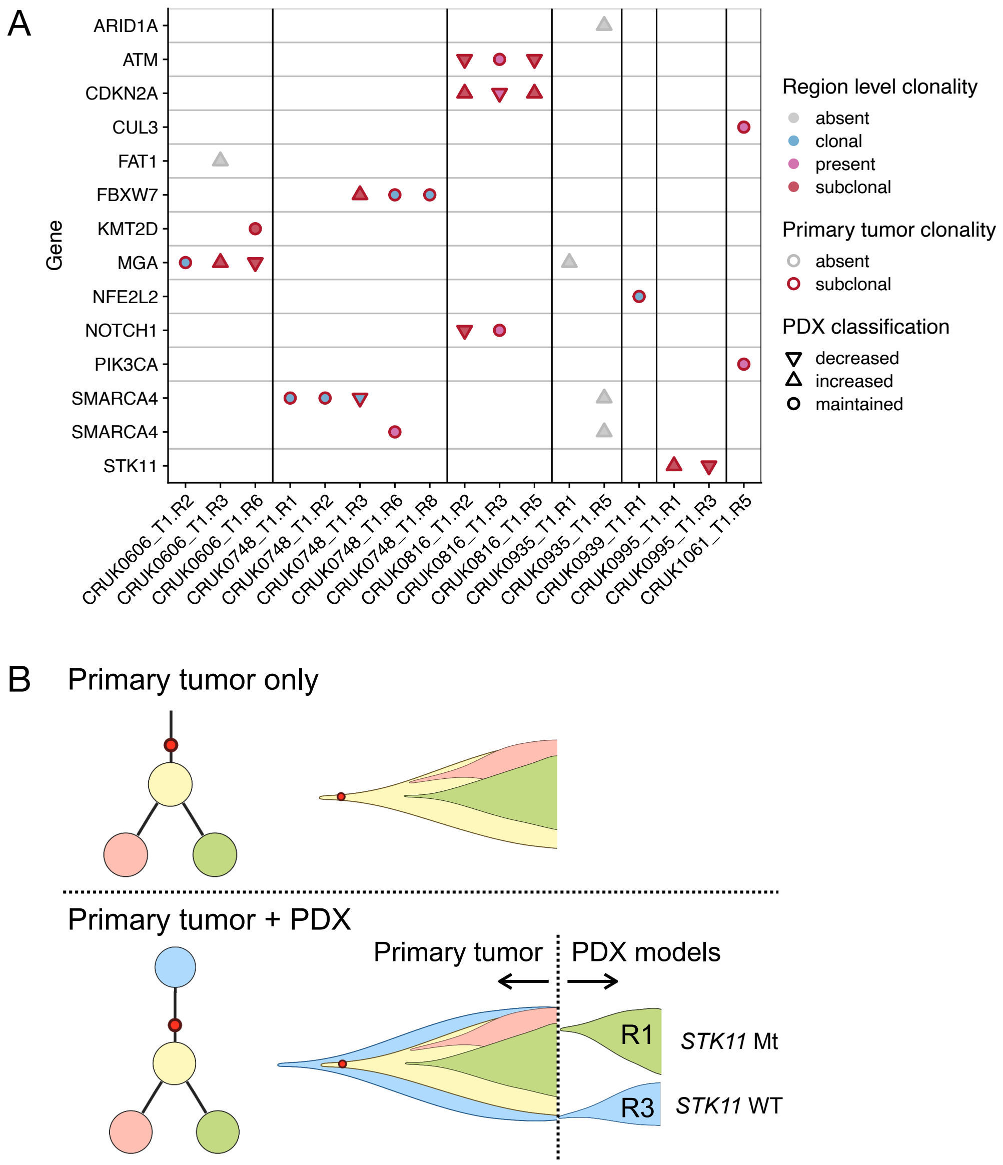

Supplementary Figure 16: Driver mutation representation in P0 PDX models compared to their matched tumor regions of origin. A) An overview of driver mutations that were subclonal (shape outline = red) or absent (shape outline = gray) in the primary tumor is shown. Each PDX model with at least one such mutation is shown and models are grouped by the primary tumor that they were derived from. The clonality of mutations in the tumor region of origin is indicated by fill color (clonal = blue, subclonal = red, present [where clonality of the mutation could not be inferred] = pink, absent = gray). Shapes indicate the relative change in clonality status in the P0 PDX model from the matched primary region of origin (circle = clonality is maintained, i.e. clonal-clonal; up arrow = clonality is increased, i.e. absent-subclonal, absent-clonal, or subclonal-clonal; down arrow = clonality is decreased, i.e. clonal-subclonal, clonal-absent, or subclonal-absent). B) Schematic of phylogenetic trees (left) and fish plots (right) showing the genomic architecture of CRUK0995 considering the TRACERx primary tumor region sequencing data only (upper panel) or both primary tumor region and PDX sequencing data (lower panel). A PDX model derived from a minor tumor subclone revealed that the *STK11* mutation (red circle) is non-truncal in this case.

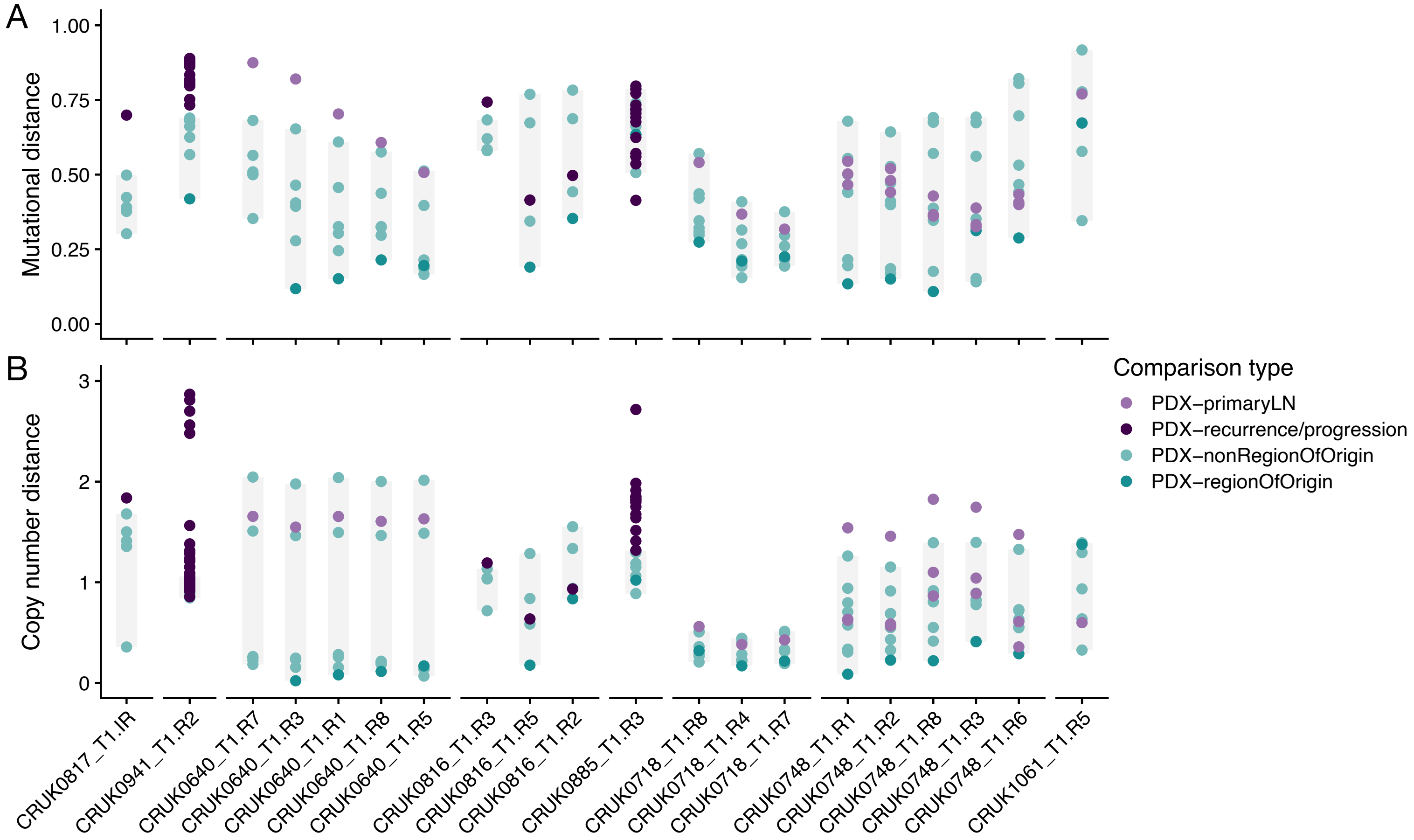

Supplementary Figure 17: Comparison of PDX models and patient metastases. Plots of A) mutational distance and B) copy number distance for samples from patients where WES data derived from metastases were available. P0 PDX models were compared to lymph node metastases at the time of primary tumor resection (light purple) or metastases sampled at post-mortem (dark purple). The distances for each PDX model with it's region of origin (dark teal) and tumor-matched non-regions of origin (teal) are shown for comparison. The total primary tumor diversity of these metrics for each PDX model is indicated by a gray box.

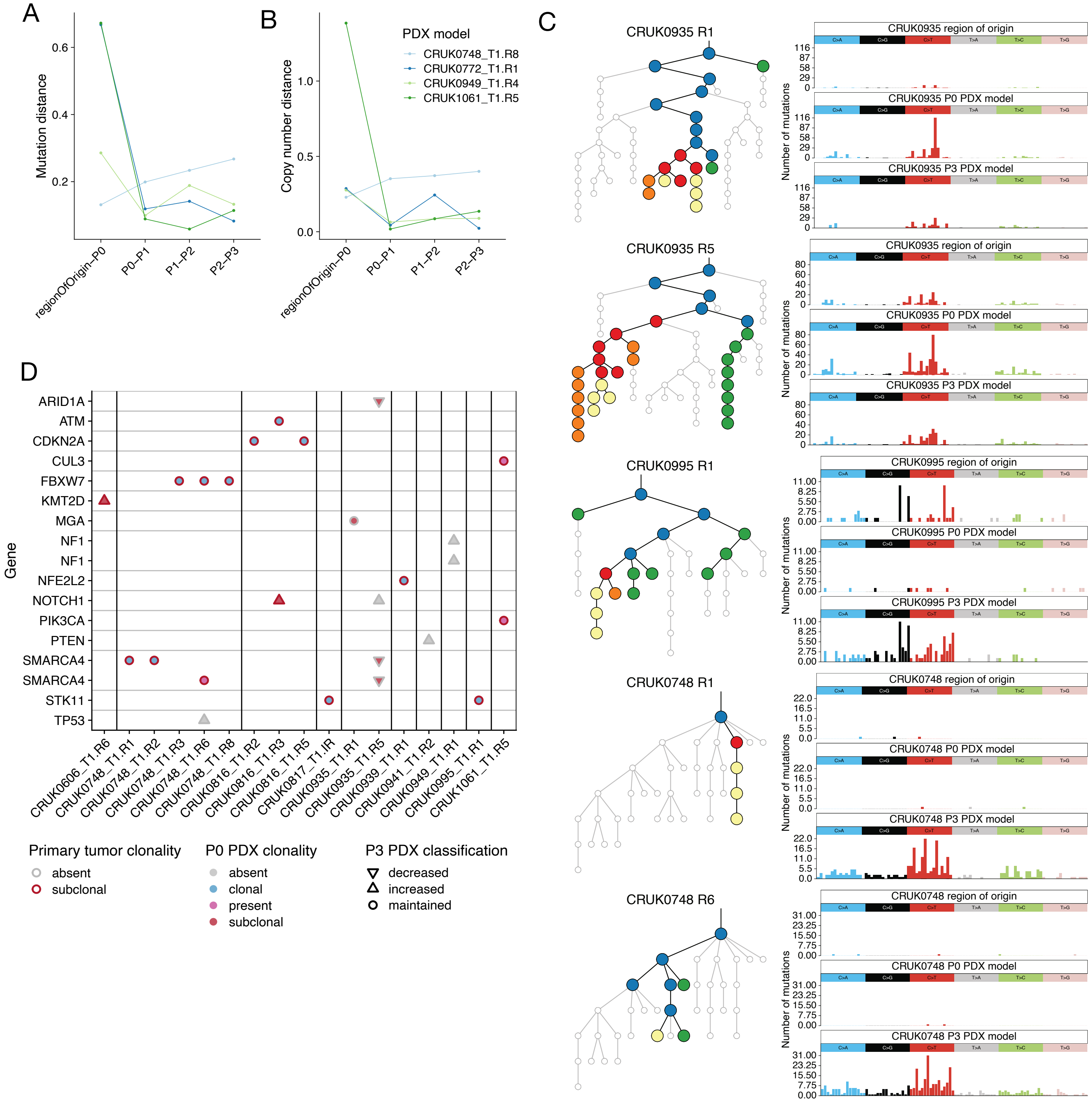

Supplementary Figure 18: On-going mutational evolution in PDX models. A) Comparison of mutational distances over sequential passage in PDX samples. B) Comparison of copy number distances over sequential passage in PDX samples. C) Phylogenetic trees (left) and mutational profiles (right) of five matched region of origin, P0 and P3 PDX models (CRUK0935 R1, CRUK0935 R5, CRUK0995 R1, CRUK0748 R1, CRUK0748 R6). The phylogenetic tree shows clusters present in the primary tumor region of origin or relevant PDX models (blue = shared between primary and PDX, green = primary specific, red = shared between P0 and P3, orange = P0 specific, yellow = P3 specific, gray = not present in the regions of interest). The mutational profiles of primary tumor unique mutations (top), P0 PDX model unique mutations (middle) and P3 PDX model unique mutations (bottom) are shown on the right of each phylogenetic tree. D) Driver mutation changes in P3 compared to P0 PDX models. An overview of driver mutations that were subclonal (shape outline = red) or absent (shape outline = gray) in the primary tumor is shown. Each PDX model with at least one such mutation is shown and models are grouped by the primary tumor that they were derived from. The clonality of mutations in the P0 PDX model is indicated by fill color (clonal = blue, subclonal = red, present [where clonality of the mutation could not be inferred] = pink, absent = gray). Shapes indicate the relative change in clonality status in the P3 PDX model from the matched P0 PDX model (circle = clonality is maintained, i.e. clonal-clonal; up arrow = clonality is increased, i.e. absent-subclonal, absent-clonal, or subclonal-clonal; down arrow = clonality is decreased, i.e. clonal-subclonal, clonal-absent, or subclonal-absent).

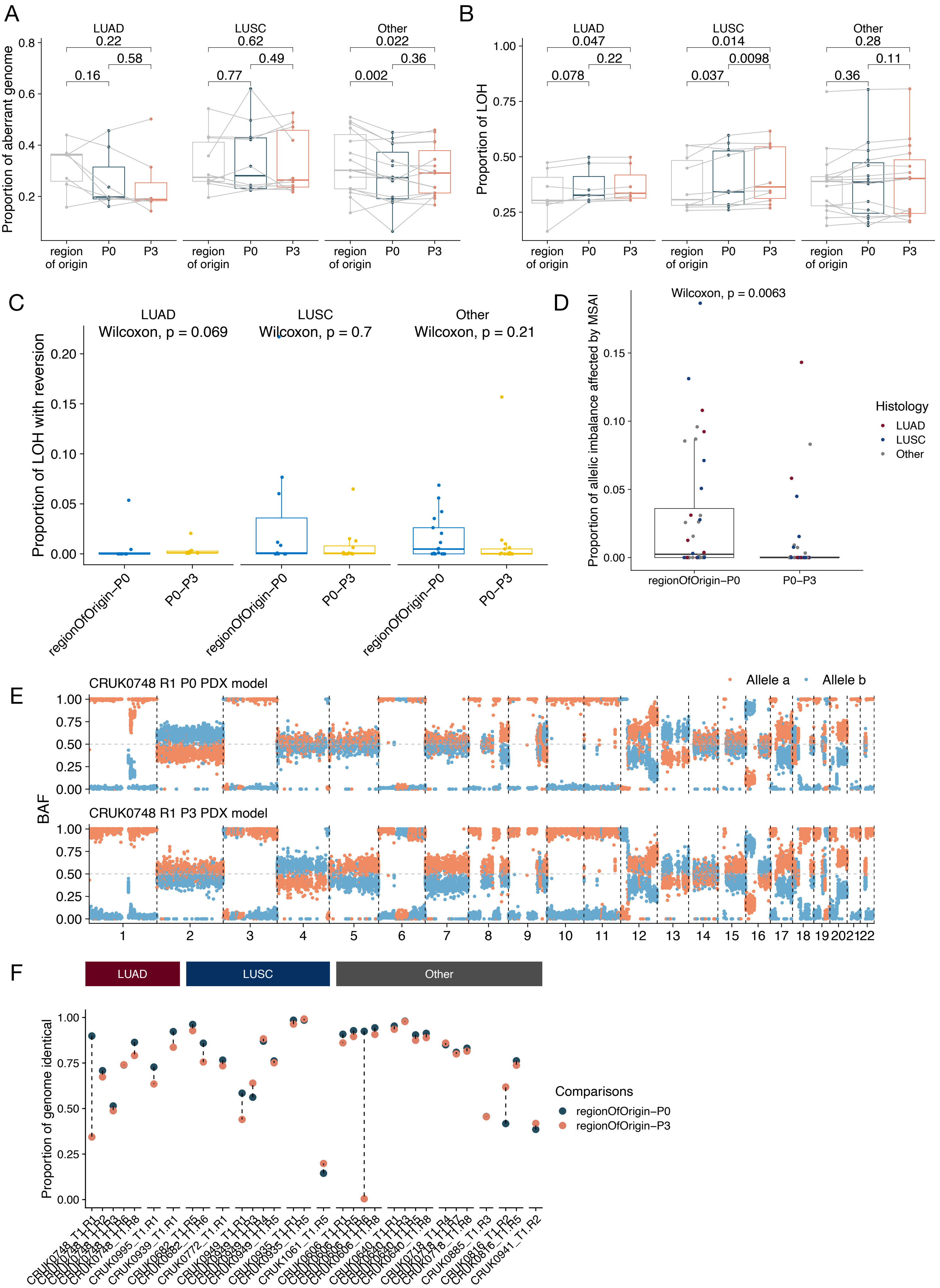
A
LUAD
0.22
0.16
0.58
LUSC
0.62
0.77
0.49
Other
0.022
0.002
0.36
Proportion of aberrant genome
0.8
0.6
0.4
0.2
region of origin
P0
P3
B
LUAD
0.047
0.078
0.22
LUSC
0.014
0.037
0.0098
Other
0.28
0.36
0.11
Proportion of LOH
1.00
0.75
0.50
0.25
region of origin
P0
P3
C
LUAD
Wilcoxon, p = 0.069
LUSC
Wilcoxon, p = 0.7
Other
Wilcoxon, p = 0.21
Proportion of LOH with reversion
0.20
0.15
0.10
0.05
0.00
regionOfOrigin−P0
P0−P3
D
Wilcoxon, p = 0.0063
Proportion of allelic imbalance affected by MSAI
0.15
0.10
0.05
0.00
regionOfOrigin−P0
P0−P3
Histology
LUAD
LUSC
Other
E
CRUK0748 R1 P0 PDX model
Allele a
Allele b
BAF
1.00
0.75
0.50
0.25
0.00
CRUK0748 R1 P3 PDX model
1 2 3 4 5 6 7 8 9 10 11 12 13 14 15 16 17 18 19 20 21 22
F
LUAD
LUSC
Other
Proportion of genome identical
1.00
0.75
0.50
0.25
0.00
Comparisons
regionOfOrigin−P0
regionOfOrigin−P3
CRUK0748_T1.R1
CRUK0995_T1.R1
CRUK0939_T1.R1
CRUK0772_T1.R1
CRUK1061_T1.R5
CRUK0885_T1.R3
CRUK0941_T1.R2

Supplementary Figure 19: On-going copy number evolution in PDX models. A) Proportion of the genome which is aberrant (either gained or lost relative to ploidy) is shown for the region of origin, P0 and P3 PDX models separated by tumor histology. Two-sided Wilcoxon signed rank test, p values as indicated. B) Proportion of the genome subject to loss of heterozygosity (LOH) is shown for the region of origin, P0 and P3 PDX models separated by tumor histology. Two-sided Wilcoxon signed rank test, p values as indicated. C) Proportion of LOH subject to reversion between region of origin and P0, as well as P0 and P3 separated by tumor histology. D) Proportion of the genome subject to mirrored subclonal allelic imbalance (MSAI) between the region of origin and P0, as well as P0 and P3. Dots are colored based on tumor histology. Two-sided Wilcoxon rank sum test, p value as indicated. E) Phased B-allele frequency plot for CRUK0748 R1 P0 (top) and P3 (bottom) PDX models. Red dots are characterized as allele A, while blue dots are characterized as allele B. F) Proportion of the genome identical between region of origin and P0 (blue) and region of origin and P3 (pink) are indicated for each PDX model where whole-exome sequencing data were available. PDX model IDs are indicated on the x-axis and are split by tumor histology. The box plots represent the upper and lower quartiles (box limits), the median (center line) and the whiskers span 1.5*IQR. LUAD - lung adenocarcinoma; LUSC - lung squamous cell carcinoma; CN - copy number.
