# Supplementary material for: Representation of genomic intratumor heterogeneity in multi-region non-small cell lung cancer patient-derived xenograft models
Source: Nat Commun. 2024 May 31;15:4653. doi: 10.1038/s41467-024-47547-3 (PMC11143323; doi:10.1038/s41467-024-47547-3)
Supplement: Supplementary file 3 — Description of Additional Supplementary Files [file 41467_2024_47547_MOESM3_ESM.docx]

**Description of Additional Supplementary Files**

Supplementary Data 1

Description:

Histological comparison of patient tumor regions and matched PDX models. Related to Supplementary Figure 5A. The findings of a comprehensive pathology review of patient tumor regions and matched PDX models. Tumors were classified on a three-point scale: 0/inconsistent = substantial differences that might affect the histopathological subtyping of the tumor; 1/divergent = minor differences affecting, for example, the growth pattern present within the samples; 2/consistent = samples are consistent. NA represents cases where one or more sample was unavailable for analysis.
