# Supplementary material for: Representation of genomic intratumor heterogeneity in multi-region non-small cell lung cancer patient-derived xenograft models
Source: Nat Commun. 2024 May 31;15:4653. doi: 10.1038/s41467-024-47547-3 (PMC11143323; doi:10.1038/s41467-024-47547-3)
Supplement: Supplementary file 5 — Reporting Summary [file 41467_2024_47547_MOESM5_ESM.pdf]

nature portfolio

Corresponding author(s): Robert E. Hynds
Nicholas McGranahan
Charles Swanton

Last updated by author(s): Feb 14, 2024



# Reporting Summary

Nature Portfolio wishes to improve the reproducibility of the work that we publish. This form provides structure for consistency and transparency in reporting. For further information on Nature Portfolio policies, see our Editorial Policies and the Editorial Policy Checklist.

## Statistics

For all statistical analyses, confirm that the following items are present in the figure legend, table legend, main text, or Methods section.

| n/a | Confirmed | |
|---|---|---|
| ☐ | ☒ | The exact sample size (*n*) for each experimental group/condition, given as a discrete number and unit of measurement |
| ☐ | ☒ | A statement on whether measurements were taken from distinct samples or whether the same sample was measured repeatedly |
| ☐ | ☒ | The statistical test(s) used AND whether they are one- or two-sided<br>*Only common tests should be described solely by name; describe more complex techniques in the Methods section.* |
| ☐ | ☒ | A description of all covariates tested |
| ☐ | ☒ | A description of any assumptions or corrections, such as tests of normality and adjustment for multiple comparisons |
| ☐ | ☒ | A full description of the statistical parameters including central tendency (e.g. means) or other basic estimates (e.g. regression coefficient) AND variation (e.g. standard deviation) or associated estimates of uncertainty (e.g. confidence intervals) |
| ☐ | ☒ | For null hypothesis testing, the test statistic (e.g. *F*, *t*, *r*) with confidence intervals, effect sizes, degrees of freedom and *P* value noted<br>*Give P values as exact values whenever suitable.* |
| ☒ | ☐ | For Bayesian analysis, information on the choice of priors and Markov chain Monte Carlo settings |
| ☒ | ☐ | For hierarchical and complex designs, identification of the appropriate level for tests and full reporting of outcomes |
| ☐ | ☒ | Estimates of effect sizes (e.g. Cohen's *d*, Pearson's *r*), indicating how they were calculated |

*Our web collection on statistics for biologists contains articles on many of the points above.*

## Software and code

Policy information about availability of computer code

| | |
|---|---|
| Data collection | PDX growth curves and PDX lineage relationships were monitored using PDX-Tracker which was developed within this manuscript and is reported here for the first time (code available at https://github.com/EpiCENTR-Lab/PDX-Tracker). |
| Data analysis | Prism (version 9.2.0)<br>R (version 4.2.2 & version 4.0.0)<br><br>Alignment and QC:<br>FastQC (version 0.11.8)<br>FastQ Screen (version 0.13.0)<br>fastp (version 0.20.0)<br>bwa-mem (version 0.7.17)<br>Sambamba (version 0.7.0)<br>Picard Tools (version 2.21.9)<br>GATK (version 3.8.1, version 4.2.0.0)<br>Somalier (version 0.2.7)<br>Samtools (version 1.9)<br>Conpair (version 0.2)<br>bamcmp (version 2.1)<br><br>NSG-adapted reference genome: (see also Zenodo https://zenodo.org/doi/10.5281/zenodo.10304174)<br>Nextflow (v21.01.4) |

nf-core Sarek pipeline (v2.7.1)
BCFtools (v1.12)

Somatic mutation and copy number calling:
SAMtools (version 1.10)
VarScan2 (version 2.4.4)
MuTect (version 1.1.7)
bam-readcount (version 0.8.0)
platypus (version 0.8.1)
ASCAT (version 2.3)
Sequenza (version 2.1.2)

refphase (version 0.3.2)
CONIPHER (version 2.2)
ParallelGDDetect (https://github.com/amf71/ParallelGDDetect)
TcellExTRECT (https://github.com/McGranahanLab/TcellExTRECT)

Independent validation pipeline:
Cutadapt (version 4.4)
bwa-mem (version 0.7.17)
bamcmp (version 2.2)
samtools (version 1.17)
Picard tools (version 3.0.0)
GATK (version 4.4.0.0)
gnomAD (version 2.1.1)
VEP (version 106)
vcf2maf (version 1.6.18)

R packages used in version 4.2.2:
ggpubr (version 0.5.0)
gridExtra (version 2.3)
gtable (version 0.3.1)
MetBrewer (version 0.2.0)
RColorBrewer (version 1.1-3)
cowplot (version 1.1.1)
lubridate (version 1.9.3)
forcats (version 1.0.0)
stringr (version 1.5.0)
dplyr (version 1.1.2)
purrr (version 1.0.2)
readr (version 2.1.4)
tidyr (version 1.3.0)
tibble (version 3.2.1)
ggplot2 (version 3.4.4)
tidyverse (version 2.0.0)
BSgenome.Hsapiens.UCSC.hg19 (version 1.4.3)
BSgenome (version 1.66.2)
rtracklayer (version 1.58.0)
Biostrings (version 2.66.0)
XVector (version 0.38.0)
ComplexHeatmap (version 2.15.4)
GenomicRanges (version 1.50.2)
GenomeInfoDb (version 1.34.4)
IRanges (version 2.32.0)
S4Vectors (version 0.36.1)
BiocGenerics (version 0.44.0)
deconstrucSigs (version 1.9.0)
data.table (version 1.14.6)
vcfR (version 1.14.0)

cloneMap  (version 1.0.0)
CONIPHER (version 2.2)

R packages used in version 4.0.0:
edgeR (version 3.32.0)
fgsea (version 1.16.0)

Histology overview figures were generated from digital pathology images using PATHOverview (code available at https://github.com/EpiCENTR-Lab/PATHOverview). PDX growth curves and PDX lineage relationships were monitored using PDX-Tracker (code available at https://github.com/EpiCENTR-Lab/PDX-Tracker).

For manuscripts utilizing custom algorithms or software that are central to the research but not yet described in published literature, software must be made available to editors and reviewers. We strongly encourage code deposition in a community repository (e.g. GitHub). See the Nature Portfolio guidelines for submitting code & software for further information.

# Data

Policy information about availability of data

All manuscripts must include a data availability statement. This statement should provide the following information, where applicable:

- Accession codes, unique identifiers, or web links for publicly available datasets
- A description of any restrictions on data availability
- For clinical datasets or third party data, please ensure that the statement adheres to our policy

The whole-exome sequencing data (primary tumor data from the TRACERx study and PDX models data) used during this study have been deposited with the European Genome–phenome Archive (EGA), which is hosted by The European Bioinformatics Institute (EBI) and the Centre for Genomic Regulation (CRG) under study accession code EGAS00001007364 and dataset accession code EGAD00001012228. Access is controlled by the TRACERx data access committee and details regarding applications for access are available on the relevant EGA page. NSG mouse whole-genome sequencing data have been deposited with The European Nucleotide Archive (ENA) and are available under the accession code PRJEB65917. The processed data, including single nucleotide polymorphisms of the NSG mouse and the NSG-adapted mouse reference genome, as well as code to reproduce these are available via Zenodo (https://zenodo.org/doi/10.5281/zenodo.10304174)68. The GRCm38/mm10 genome assembly can be downloaded from UCSC (https://hgdownload.soe.ucsc.edu/goldenPath/mm10/bigZips/).

Biological materials, including PDX models generated within this study, are available to the community for academic non-commercial research purposes via standard MTA agreements.

# Research involving human participants, their data, or biological material

Policy information about studies with human participants or human data. See also policy information about sex, gender (identity/presentation), and sexual orientation and race, ethnicity and racism.

| | |
|---|---|
| Reporting on sex and gender | Sex of participants is recorded within the study and has been reported within Figure 1B, Supplementary Figure 1A and the clinical data file available on Zenodo. Information on patients' gender was not collected as part of the study. |
| Reporting on race, ethnicity, or other socially relevant groupings | Data on patients' ethnicity was collected and has been reported in Supplementary Figure 1F and the clinical data file available on Zenodo. |
| Population characteristics | For the TRACERx 421 cohort that is used within the study, 421 patients are included in this TRACERx cohort. 44.6% are females , 55.4% males; 93% are smokers of have a smoking history, 7% are never smokers; 25% of patients were diagnosed at stage IA, 25% at IB, 17.8% at IIA, 13.5% at IIB, 18.5% at IIIA and 0.2% at IIIB; 52% of diagnosed tumours were adenocarcinomas, 28.8% were squamous cell carcinomas and 19.2% were of other histological subtypes; 93% of the cohort is from a white ethnic background and the mean age of the patients is 69, ranging between 34 and 92.<br><br>The PDX model cohort is obtained from a subset of TRACERx patients, partially overlapping with the TRACERx421 cohort. No significant differences in clinical characteristics were observed and this data is reported in Supplementary Figure 1. |
| Recruitment | When patients are initially diagnosed with stage I-III lung cancer and then referred for surgical resection, a research nurse identifies them on a clinic/operating list. The patient has an initial eligibility assessment and then provided with written information about the TRACERx study and he/she can ask the research nurse any questions.<br><br>Patients have to agree to provide serial blood samples whenever they attend clinic for routine blood sampling, so this represents the only main potential self-selecting bias (i.e. only patients willing to do this would participate). However, it is unclear how this would affect the biomarker analyses. Also, the gender and ethnicity characteristics are in line with patients seen in routine practice.<br><br>Inclusion Criteria:<br>_Written Informed consent<br>_Patients ≥18 years of age, with early stage I-IIIB disease (according to TNM 8th edition) who are eligible for primary surgery.<br>_Histopathologically confirmed NSCLC, or a strong suspicion of cancer on lung imaging necessitating surgery (e.g. diagnosis determined from frozen section in theatre)<br>_Primary surgery in keeping with NICE guidelines planned<br>_Agreement to be followed up at a TRACERx site<br>_Performance status 0 or 1<br>_Minimum tumor diameter at least 15mm to allow for sampling of at least two tumour regions (if 15mm, a high likelihood of nodal involvement on pre-operative imaging required to meet eligibility according to stage, i.e. T1N1-3)<br><br>Exclusion Criteria:<br>_Any other* malignancy diagnosed or relapsed at any time, which is currently being treated (including by hormonal therapy).<br>_Any other* current malignancy or malignancy diagnosed or relapsed within the past 3 years**.<br>*Exceptions are: non-melanomatous skin cancer, stage 0 melanoma in situ, and in situ cervical cancer<br>**An exception will be made for malignancies diagnosed or relapsed more than 2, but less than 3, years ago only if a pre-operative biopsy of the lung lesion has confirmed a diagnosis of NSCLC.<br>_Psychological condition that would preclude informed consent<br>_Treatment with neo-adjuvant therapy for current lung malignancy deemed necessary<br>_Post-surgery stage IV<br>_Known Human Immunodeficiency Virus (HIV), Hepatitis B Virus (HBV), Hepatitis C Virus (HCV) or syphilis infection. |

_Sufficient tissue, i.e. a minimum of two tumor regions, is unlikely to be obtained for the study based on pre-operative imaging

Patient ineligibility following registration
_There is insufficient tissue
_The patient is unable to comply with protocol requirements
_There is a change in histology from NSCLC following surgery, or NSCLC is not confirmed during or after surgery.
_Change in staging to IIIC or IV following surgery
_The operative criteria are not met (e.g. incomplete resection with macroscopic residual tumors (R2)). Patients with microscopic residual tumors (R1) are eligible and should remain in the study
_Adjuvant therapy other than platinum-based chemotherapy and/or radiotherapy is administered.

Ethics oversight

The study was approved by the NRES Committee London with the following details:
Study title: TRAcking non small cell lung Cancer Evolution through therapy (Rx)
REC reference: 13/LO/1546
Protocol number: UCL/12/0279
IRAS project ID: 138871

Note that full information on the approval of the study protocol must also be provided in the manuscript.

# Field-specific reporting

Please select the one below that is the best fit for your research. If you are not sure, read the appropriate sections before making your selection.

☒ Life sciences ☐ Behavioural & social sciences ☐ Ecological, evolutionary & environmental sciences

For a reference copy of the document with all sections, see nature.com/documents/nr-reporting-summary-flat.pdf

# Life sciences study design

All studies must disclose on these points even when the disclosure is negative.

Sample size

TRACERx is a programme of work of multiple projects built around a single observational cohort study. It is not possible to perform a sample size calculation for each project, especially post hoc. The study size of the cohort was done in relation to tumour heterogeneity and disease free survival:

The overall study sample size is based on demonstrating a relationship between tumours with divergent intratumour heterogeneity index values and clinical outcome. Patients will be split evenly into those with a low and high intratumour heterogeneity index value (and other splits will be considered). Assuming a median Disease Free Survival (DFS) of 30 months and a hazard ratio (HR) of 0.77, with a 2-sided 5% significance level, 90% power, accrual period of 3 years and 5 years follow-up after the end of accrual, the sample size required is almost 400 per group (total of 800 patients). Assuming a 5% dropout rate, a total of 842 patients (421 per group) are required. At 85% power, 705 patients would be required in total, which could be the minimum target. However, we will instead aim for 750 patients and recruitment will continue for the length of time which is funded for accrual in order to get as close as possible to the ideal target of 842 patients. A study size of 842 is also large enough to detect a 10% improvement in a 5 year OS rate from 46% in the high Intratumour Heterogeneity Index (ITB) to 56% in the low Intratumour Heterogeneity Index group (HR=0.75), with 80% power and a 2 sided type I error set at 5% (logrank test). A high/low ITB value will be defined as values above/below the 50th percentile (median ITB). We have a target DFS effect of a 23% reduction in risk (hazard ratio 0.77), which means that our study is powered for an effect at least this large, including a 30% difference (which has been the target for progression-free survival in trials of advanced NSCLC, in relation to expected effects on OS).

Data exclusions

Please see study inclusion/exclusion criteria below. Additionally, samples which fail quality control metrics were also excluded from analysis.

Replication

TRACERx is a prospective longitudinal study. The results shown here represent the latest data from a prospective PDX derivation pipeline. In future there might be the opportunity to develop a validation cohort with further samples. In a limited number of patients, we report replicate PDX model lineages that derive from the same P0 PDX tumor - the sequencing results of these experiments were consistent with our main histological and sequencing findings. For findings comparing the use of an NSG-adapted reference genome to the standard mm10 reference genome, we replicated our findings in an independent cohort of PDX models using an independent bioinformatics pipeline at the University of Manchester.

Randomization

No randomization was performed in the this study as no therapeutic interventions are tested.

Blinding

No blinding was performed in this study as user knowledge of patient or tumor characteristics could not have affected outcomes (i.e. genetic similarity of PDX models to their parent tumor).

# Reporting for specific materials, systems and methods

We require information from authors about some types of materials, experimental systems and methods used in many studies. Here, indicate whether each material, system or method listed is relevant to your study. If you are not sure if a list item applies to your research, read the appropriate section before selecting a response.

## Materials & experimental systems

| n/a | Involved in the study |
| --- | --- |
| ☐ | ☒ Antibodies |
| ☒ | ☐ Eukaryotic cell lines |
| ☒ | ☐ Palaeontology and archaeology |
| ☐ | ☒ Animals and other organisms |
| ☐ | ☒ Clinical data |
| ☒ | ☐ Dual use research of concern |
| ☒ | ☐ Plants |

## Methods

| n/a | Involved in the study |
| --- | --- |
| ☒ | ☐ ChIP-seq |
| ☒ | ☐ Flow cytometry |
| ☒ | ☐ MRI-based neuroimaging |

# Antibodies

| | |
| --- | --- |
| Antibodies used | anti-CD45 (Clone HI30; Dilution 1:200; Cat No 304002); anti-keratin (Clone: AE1/AE3; Dilution: 1:100; Cat No: 13160); anti-CD3 (Clone: LN10; Dilution: 1:100; Cat No: NCL-L-CD3-565); anti- CD20 (Clone L26; Dilution: 1:200; Cat No: M0755) |
| Validation | Optimization of the antibodies was carried out on sections of human tonsil tissues, with assessment by an experienced consultant pathologist. |

# Animals and other research organisms

Policy information about studies involving animals; ARRIVE guidelines recommended for reporting animal research, and Sex and Gender in Research

| | |
| --- | --- |
| Laboratory animals | Male non-obese diabetic/severe combined immunodeficient (NOD/SCID/IL2Rg-/-; NSG) mice were housed in individually ventilated cages under specific pathogen-free conditions and had ad libitum access to sterile food and water, The room housing the mice was maintained on a 12 hour light-dark cycle (with gradually increasing light from 6:30am-7:00am and gradually decreasing light from 6:30-7:00pm). Temperature was maintained in a 20-24°C range and humidity was maintained at 55% (+/- 10%). Mice were typically between 6 and 12 weeks of age at the time of tumor/PDX implantation. |
| Wild animals | N/A |
| Reporting on sex | Male mice were used in the study and this is reported in the Methods section. |
| Field-collected samples | No field collected samples were used in the study. |
| Ethics oversight | Animal studies were approved by the University College London Biological Services Ethical Review Committee and licensed under UK Home Office regulations (P36565407). This sentence is included in the methods section. |

Note that full information on the approval of the study protocol must also be provided in the manuscript.

# Clinical data

Policy information about clinical studies
All manuscripts should comply with the ICMJE guidelines for publication of clinical research and a completed CONSORT checklist must be included with all submissions.

| | |
| --- | --- |
| Clinical trial registration | TRACERx lung https://clinicaltrials.gov/ct2/show/NCT01888601, approved by an independent Research Ethics Committee, 13/LO/1546 |
| Study protocol | https://clinicaltrials.gov/ct2/show/NCT01888601 |
| Data collection | Clinical and pathological data is collected from patients during study follow up - this period is a minimum of five years. Data collection is overseen by the sponsor of the study (Cancer Research UK & UCL Cancer Trials Centre) and takes place in hospitals across the United Kingdom. A centralised database called MACRO is used for this purpose. Recruitment started in April 2014 and finished in 2023. |
| Outcomes | The main clinical outcome is disease-free survival (DFS). DFS was measured from the time of study registration to date of first lung recurrence or death from any cause. Patients who do not have these events are censored at the date last known to be alive (including patients who developed a new primary tumour that has been shown biologically to not be linked to the initial primary lung tumour). |

## Plants

| | |
|---|---|
| Seed stocks | N/A |
| Novel plant genotypes | N/A |
| Authentication | N/A |
